# Modifying Role of Sustainable Diets on the Association Between Particulate Matter and Biological Aging: The Guangzhou Biobank Cohort Study

**DOI:** 10.1111/acel.70422

**Published:** 2026-02-27

**Authors:** Rui Qiang Li, Shou Xin Peng, Rui Hang Zhang, Ting Yu Lu, Wei Sen Zhang, Jiao Wang, Ying Wang, Lin Yang, Shiu Lun Ryan Au Yeung, Tai Hing Lam, Kar Keung Cheng, Lin Xu

**Affiliations:** ^1^ School of Public Health Sun Yat‐Sen University Guangzhou China; ^2^ Central China Subcenter of National Center for Cardiovascular Diseases, Henan Cardiovascular Disease Center, Fuwai Central‐China Cardiovascular Hospital Central China Fuwai Hospital of Zhengzhou University Zhengzhou China; ^3^ Guangzhou Twelfth People's Hospital Guangzhou China; ^4^ Great Bay Area Public Health Research Collaboration Guangzhou Guangdong China; ^5^ School of Nursing The Hong Kong Polytechnic University Hong Kong China; ^6^ School of Public Health The University of Hong Kong Hong Kong China; ^7^ Institute of Applied Health Research University of Birmingham Birmingham UK

**Keywords:** planetary‐health diet score, plant‐based diet index, PM_2.5_ and PM_10_, polygenic risk score, relative telomere length

## Abstract

Air pollution accelerates biological aging via oxidative stress and inflammation, a process potentially mitigated by plant‐based diets. However, the role of dietary or genetic modulators in this relationship remains understudied. Our study aimed to examine whether adherence to Sustainable Diets modifies the associations of PM_2.5_ and PM_10_ exposure with biological aging, and to assess potential effect modification by genetic susceptibility to longevity. Data from 9527 participants in the Guangzhou Biobank Cohort Study were used to examine the association of one‐year average PM_2.5_ and PM_10_ exposure with biological aging, measured by phenotypic age, accelerated age, and relative telomere length (RTL). Sustainable diet adherence was assessed using Plant‐Based Diet Index (PDI) and Planetary Health Diet (PHD) scores, with higher scores indicating greater adherence. The mean age of participants was 64.6 years (SD = 6.0). Higher exposure to PM_2.5_ (*β* = 0.039) and PM_10_ (*β* = 0.028) was associated with increased phenotypic age and higher odds of accelerated aging (OR = 1.008 for PM_2.5_, OR = 1.005 for PM_10_). These associations weakened with greater adherence to a sustainable diet (higher PDI/PHD scores). Stronger associations were found in those with lower polygenic risk scores for longevity. A suggestive association between higher PM exposure and shorter RTL was observed, particularly in participants ≥ 65 years and those with cardiovascular diseases. Sustainable dietary patterns rich in plant‐based foods may attenuate the associations between air pollution and biological aging in older adults. These findings highlight the modifying role of dietary patterns as a potential strategy to mitigate the pollution‐related aging burden.

## Graphical abstract

Higher exposure to PM_2.5_ and PM_10_ accelerates biological aging, a process significantly mitigated by adherence to sustainable, plant‐based dietary patterns. These results underscore the potential of sustainable diets to counteract the detrimental effects of air pollution on healthy aging and longevity.

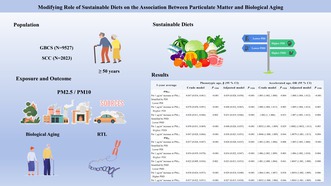

## 1 Introduction

Air pollution is a major global public health threat, responsible for approximately 7 million deaths annually according to the World Health Organization, with older adults particularly vulnerable. Prolonged exposure to particulate matter (PM_2.5_ and PM_10_) accelerates deoxyribonucleic acid (DNA) methylation age and urinary peptide clock (Schmidt 2024; Ward‐Caviness et al. 2016), shortens telomere length (Wong et al. 2014; Wu et al. 2023), and increases levels of high‐sensitivity C‐reactive protein, interleukin‐6, and fibrinogen (Chuang et al. 2007; Pritha et al. 2024). Collectively, these changes contribute to accelerated biological aging, reduced quality of life, and shortened life expectancy.

While numerous epidemiological studies have linked air pollution exposure to biological aging, most have focused on direct health outcomes without considering the potential mitigating effects of dietary patterns (Assavanopakun et al. 2022; Baranyi et al. 2022; Martens et al. 2023). Dietary habits have significant potential for mitigating the adverse impacts of air pollution. Sustainable dietary patterns, particularly those emphasizing plant‐based foods and aligned with planetary health diets (PHD), are characterized by high antioxidants, vitamins, minerals, and fiber content (Satija and Hu 2018; Shaw et al. 2022), nutrients known to reduce oxidative stress and inflammation, key pathways activated by air pollution (Myhrstad and Wolk 2023). The PHD, designed to balance nutritional adequacy with environmental sustainability, aligns with global health goals aimed at achieving harmony between human and planetary well‐being (Springmann et al. 2016; Willett et al. 2019).

As the fifth leading risk factor for global mortality, air pollution contributes to worldwide disease burden across diverse populations (Cohen et al. 2017; Li et al. 2018; Liu et al. 2022; Strak et al. 2021). Notably, previous studies from different settings showed that adherence to both the PHD and plant‐based diet index (PDI) lowered the risks of diabetes (Satija et al. 2016), coronary heart disease (Satija et al. 2017), cognitive decline (Lee et al. 2017; van Soest et al. 2024), cancer (Karavasiloglou et al. 2023), and all‐cause mortality (Chen et al. 2024; Thompson et al. 2023). Global promotion of PHD could prevent approximately 15 million premature deaths annually, accounting for 27% of global mortality (Gu et al. 2024). Plant‐based diets may also mitigate oxidative stress and inflammation induced by air pollution, reducing cardiovascular disease risk (Lim et al. 2019; Serafini and Peluso 2016). Additionally, higher adherence to the plant‐based diets has been associated with slower cognitive decline due to long‐term PM_2.5_ exposure (Zhu et al. 2022), suggesting the potential role of dietary antioxidants against pollution‐related cognitive impairment (Robinson 2017; Schulz et al. 2015).

Despite these promising associations, critical evidence gaps remain. The PDI primarily targets individual health with incidental sustainability benefits (Pimentel and Pimentel 2003), and the PHD is explicitly designed to promote human‐environmental synergy. Whether adherence to these sustainable dietary patterns modifies the adverse association between air pollution and biological aging has not been well explored. Existing studies have largely examined air pollution and aging in isolation, with limited attention to potentially modifiable dietary factors or to effect modification by genetic susceptibility. We therefore examined interactions between sustainable diet and air pollution in relation to the biological aging process, and further assessed effect modification by genetic predisposition to longevity using a polygenic risk score (PRS).

## 2 Materials and Methods

### 2.1 Study Population

The Guangzhou Biobank Cohort Study (GBCS) is a three‐way collaboration involving the Guangzhou Twelfth People's Hospital, the University of Hong Kong, and the University of Birmingham in the UK (Jiang et al. 2006). Participants were recruited from the Guangzhou Health and Happiness Association for the Respectable Elders (GHHARE). GHHARE is unofficially aligned with the municipal government. Membership is open to local permanent residents aged 50+ years with a nominal fee of 4 RMB (≈50 US cents) per month. GHHARE included about 7% of Guangzhou residents in this age group, with branches in all districts of Guangzhou, the capital city of Guangdong Province in southern China (Jiang et al. 2006).

Baseline data were collected through face‐to‐face interviews using computer‐assisted questionnaire, conducted by trained nurses from the Guangzhou Twelfth People's Hospital. This data collection encompassed demographic information, lifestyle factors, family and personal medical histories, and anthropometric measurements, all adhering to a standardized protocol. The study received approval from the Guangzhou Medical Ethics Committee of the Chinese Medical Association, and all participants provided written informed consent before participation. As shown in the participant flow diagram (Figure S1), a total of 10,413 participants were initially enrolled in the GBCS during Phase 1 (2003–2004). After excluding 886 participants due to missing data on nutrients (*n* = 157), phenotypic age (*n* = 235), key covariates (*n* = 219), or PM_2.5_/PM_10_ exposure (*n* = 275), the final analytical sample consisted of 9527 individuals. These participants were followed from the baseline examination (2003–2004) until November 21, 2023, for the assessment of all‐cause mortality.

The South China Cohort (SCC) is a prospective study designed to explore the epidemiology, etiology, and risk factors of non‐communicable diseases (NCDs) in southern China (Yang et al. 2024). It employed a multi‐stage stratified cluster sampling method to recruit 116,520 permanent residents aged 25–89 from 13 cities across Guangdong, Fujian, Guangxi, and Hainan provinces from March 2018 to December 2020, ensuring a balanced representation of age and gender. Written informed consent was obtained from all participants before participation. The study was conducted in compliance with the Declaration of Helsinki and was approved by the Ethics Committee of the School of Public Health at Sun Yat‐sen University (L2017‐001). Follow‐up includes both passive and active tracking. Passive follow‐up links to the National Death Surveillance System (NDSS) and National Health Insurance Database (NHID) annually, achieving a first‐round follow‐up rate of 93.7%. Active follow‐up is conducted every 5 years on 20% of participants through face‐to‐face interviews, physical examinations, and biospecimen collection. At baseline, demographic information, health status, and additional variables were collected using standardized electronic questionnaires administered by trained staff. The surveys were completed on tablets and required about 30–40 min per participant. Physical examinations were performed by trained healthcare professionals or community health center staff using regularly calibrated instruments. For this analysis, we used data from 2023 SCC participants to examine the association between exposure to PM_2.5_ and PM_10_ and relative telomere length, measured via quantitative PCR (qPCR) as described by Cawthon (2002) and expressed as T/S ratio (telomere‐to‐single‐copy gene ratio) (Figure S2).

### 2.2 Exposure Assessment

High‐quality, high‐resolution data (1 km × 1 km) on PM_2.5_ and PM_10_ exposure for each participant were obtained from the ChinaHighAirPollutants (CHAP) dataset, which integrates multi‐source satellite remote sensing and artificial intelligence technologies (https://weijing‐rs.github.io/product.html). The dataset combines ground‐based monitoring data, satellite remote sensing products, atmospheric reanalysis, and model simulations to provide accurate exposure estimates while accounting for the spatial and temporal heterogeneity of air pollution. For PM_2.5_, the dataset demonstrated excellent predictive accuracy, with a 10‐fold cross‐validation *R* ^2^ of 0.92 and a root mean square error (RMSE) of 10.76 μg/m^3^ (Wei, Li, Lyapustin, et al. 2021). Similarly, for PM_10_, the *R* ^2^ was 0.90, with an RMSE of 21.12 μg/m^3^ (Wei, Li, Xue, et al. 2021). To ensure precise exposure assignment, participants' residential addresses were geocoded into longitude and latitude coordinates. The monthly average exposure levels of PM_2.5_ and PM_10_ were extracted from the specific 1 km grid cell corresponding to each participant's location. For the primary analysis, long‐term air pollution exposure was assessed using the average monthly concentrations of PM_2.5_ and PM_10_ during the year preceding the baseline survey. To illustrate the spatial distribution of participants' residences, we generated a map depicting their geographic locations, which is now provided as Figure S3.

### 2.3 Dietary Intake Assessment

Dietary intake was assessed at baseline using a validated Food Frequency Questionnaire (FFQ), which included 300 commonly consumed food and beverage items in Southern China. The development, validation, and related findings of this FFQ have been thoroughly described in previous studies (Sun et al. 2024; Woo et al. 1997; Xu et al. 2019). Data on dietary intake were collected through face‐to‐face interviews conducted by trained nurses, following a 1‐week period. Participants provided detailed information on all foods and beverages consumed, including portion sizes and frequency of consumption. To improve the accuracy of portion size reporting, a photo catalog of typical food portions was provided. The weekly intake of each food and beverage item was then calculated based on the portion size and frequency data collected. The validity of the FFQ was confirmed through comparative analyses: estimates of total energy, sodium, and potassium intake derived from the FFQ were compared with values obtained from established methods, such as energy expenditure measurements and 24‐h urinary sodium and potassium excretion. Furthermore, correlations between dietary cholesterol and fatty acid intake assessed by the FFQ and plasma lipid profiles were examined, further supporting the validity of the FFQ through these rigorous procedures (Woo et al. 1997).

### 2.4 Plant‐Based and Planetary‐Health Diets

The PDI was constructed using 18 food groups classified into three categories: (1) healthy plant foods, including whole grains, fruits, vegetables, nuts, legumes and vegetarian protein alternatives (i.e., plant‐based options such as tofu, textured vegetable proteins, and seitan), tea and coffee and vegetable oils; (2) unhealthy plant foods, such as fruit juices, refined grains, potatoes, sugar‐sweetened beverages, sweets and desserts; and (3) animal‐derived foods, including animal fat, dairy, eggs, fish or seafood, meat, and other miscellaneous animal‐derived foods (Satija et al. 2016; Thompson et al. 2023). Intake for each food group exceeding zero portions was categorized into quartiles, with participants ranked accordingly. Scores were assigned from 2 to 5 based on quartile ranking, with the lowest intake receiving a score of 2 and the highest intake a score of 5. Participants with zero intake were assigned a score of 1. Conversely, scoring for animal‐derived foods was inverted, with higher intake receiving lower score (Zhu et al. 2022).

The PHD score, ranging from 0 to 140, was based on the 14 key dietary recommendations of the EAT–Lancet Commission (Cacau et al. 2021; Willett et al. 2019). These components were grouped into three categories according to their health implications: adequacy, optimum, and moderation. Adequacy components included whole grains, all vegetables, fruits, legumes, and nuts. Optimum components encompassed poultry, dairy foods, eggs, fish, unsaturated oils, and tuber and starchy vegetables. Moderation components consisted of red meat, saturated oils, and added sugar. All daily food intakes were standardized to an energy intake of 2500 kcal/day. Continuous scores ranging from 0 to 10 were assigned to each food group, with higher scores indicating closer adherence to dietary recommendations. Total PHD score, summing these components, ranged from 0 (no adherence to the PHD) to 140 (complete adherence to the PHD) (Ye et al. 2023).

### 2.5 Assessment of Biological Aging

Phenotypic age, accelerated age, and all‐cause mortality were employed in the GBCS as comprehensive indicators of biological aging, while relative telomere length (T/S ratio) was used in the SCC to assess cellular‐level aging. Phenotypic age was determined using a weighted linear combination of chronological age (in years) and eight biomarkers: albumin, creatinine, glucose, C‐reactive protein, lymphocyte percentage, mean corpuscular volume, red cell distribution width, and white blood cell count (Liu et al. 2018). It was calculated based on the following formula:

$$\text{Phenotypic}\;\mathrm{age}=141.50225+\frac{\ln\left(-0.00553\times \ln\left(1-\text{score}\right)\right)}{0.090165}$$

where:

$$\text{Score}=1-e^{-e^{\mathrm{xb}}\left(\exp\left(120\times \gamma \right)-1\right)/\gamma }$$

*γ* was 0.0076927, and:

$$\begin{array}{cc} \mathrm{xb}=\; & -19.9067-0.0036\times \text{albumin}+0.0095\\ & \times \text{creatine}+0.1953\times \text{glucose}+0.0954\\ & \times \ln\left(\mathrm{CRP}\right)-0.0120\times \text{lymphocyte percent}\\ & +0.0268\times \text{mean cell volume}+0.3306\\ & \times \mathrm{red}\;\text{cell distributionwidth}+0.0554\\ & \times \text{white blood cell count}+0.0804\times \text{chronological}\;\mathrm{age} \end{array}$$

Accelerated age was defined as the residual derived from a linear regression model of phenotypic age on chronological age (Liu et al. 2018). Additionally, accelerated age was dichotomized into two categories: presence or absence of accelerated aging, with values greater than 0 indicating accelerated aging (Yang et al. 2022).

### 2.6 Genetic Data

As mentioned in our previous study (Tian et al. 2025), genomic DNA was extracted from buffy coats of 3137 participants. Genotyping was performed using the Illumina Asian Screening Array‐24 + v1.0 (ASA) platform, which assays 743,722 genetic variants. All genotype data underwent rigorous quality control (QC) prior to analysis. Sample‐level QC included checks for call rate (< 98%), heterozygosity (±3 SD from the mean), and gender discordance. Variant‐level QC involved filtering on call rate (> 97%), minor allele frequency (MAF > 0.01), and deviation from Hardy–Weinberg equilibrium (HWE *p* > 1 × 10^−4^). A detailed description of the QC procedures is provided in the Supporting Information.

### 2.7 Polygenic Risk Score

The construction of the PRS was based on a panel of candidate variants previously identified as being associated with life expectancy. Specifically, 12 single‐nucleotide polymorphisms (SNPs) were initially selected based on their validated association with longevity in the Chinese Longitudinal Healthy Longevity Survey, the methodology for which has been described in detail elsewhere (Liu et al. 2021). In the GBCS, we applied stringent QC protocols to ensure the reliability of the genetic data. Seven SNPs were excluded from the final analysis due to genotyping failure. Consequently, the final PRS was constructed using five SNPs (rs16981095, rs3803304, rs1043943, rs2075650, and rs11925757) that passed all QC filters. Detailed information is provided in Table S1. The PRS was calculated as a weighted sum of effect alleles using published effect estimates (Liu et al. 2021; Tian et al. 2025). A lower PRS indicates a reduced genetic predisposition for longevity, suggesting fewer beneficial genetic variants associated with extended lifespan or a higher risk of early mortality.

### 2.8 Covariates

The covariates included in this study encompassed a broad spectrum of demographic, socioeconomic, lifestyle, environmental, and health factors. Demographic factors included age (< 65 years, ≥ 65 years) and gender (female, male). Socioeconomic status was assessed through education level (primary or below, secondary, college or above), occupation (manual, non‐manual, other), and family income (< 10,000, 10,000–29,999, 30,000–49,999, ≥ 50,000 CNY/year, don't know). Lifestyle variables included smoking status (never, former, current), alcohol consumption (never, former, current), physical activity level (inactive, moderate, active), and body mass index (BMI, < 18.5, 18.5–24.9, 25.0–27.4, ≥ 27.5 kg/m^2^). Environmental factors were represented by household air pollution exposure (yes, no), passive smoking exposure (< 2 years of 40 h per week, 2–5 years of 40 h per week, > 5 years of 40 h per week, not reported), temperature (°C), humidity (%), and ozone (μg/m^3^). Health status was captured by the presence of arthritis (yes, no), diabetes (yes, no), hypertension (yes, no), dyslipidemia (yes, no), and cardiovascular disease (yes, no), chronic obstructive pulmonary disease (COPD, yes, no) (Figure S4).

### 2.9 Statistical Analysis

Characteristics of the study population were described using frequency (percentage) for categorical variables, and mean ± standard deviation (SD) and median [interquartile range (IQR)] for continuous variables. Here, we utilize the Shapley interaction index to capture local interactions. The Shapley interaction index is more novel than the classical concept of Shapley values and is derived from the generalization of the properties of the original Shapley values (Lundberg et al. 2020). SHapley Additive exPlanations (SHAP) interaction plots were used to explore potential non‐linear interactions between long‐term exposure to PM_2.5_ and PM_10_ and sustainable diet adherence, as assessed by PDI and PHD scores, in their associations with biological aging metrics. Subgroup analyses were performed by stratifying participants into high and low sustainable diet score groups based on their PHD and PDI to evaluate the potential modifying role of dietary adherence in the association between air pollution exposure and biological aging. To establish a temporal sequence, air pollution exposure was estimated using the average annual concentration in the year preceding baseline data collection. Dietary intake was assessed via FFQ, reflecting the previous 7 days, while biological aging markers were measured at the time of the survey. This design ensured that exposure and covariate data preceded the biological aging outcomes.

Linear regression models were used to estimate *β* coefficients and 95% confidence intervals (CIs) for phenotypic age, while logistic regression models were used to estimate odds ratios (ORs) and 95% CIs for accelerated age. Crude and adjusted models were used, adjusting for potential confounders including age, gender, education, occupation, family income, smoking status, alcohol use, BMI, physical activity, household air pollution, passive smoking exposure, temperature, humidity, and O_3_, arthritis, diabetes, hypertension, dyslipidaemia, COPD, and cardiovascular disease. Additionally, three‐knot cubic splines (with knots placed at the 10th, 50th, and 90th percentiles) were used to examine potential non‐linear relationships between air pollution exposure and aging outcomes. The choice of three knots was guided by the principle of parsimony, aiming to ensure adequate model flexibility while minimizing the risk of overfitting (Discacciati et al. 2025; Nguyen et al. 2020). This was further supported by the lowest Akaike Information Criterion among the candidate models. To further evaluate the modifying effect of genetic susceptibility, an interaction term between the PM and PRS was included in the regression models, and subgroup analyses were conducted based on lower and higher PRS categories. Stratified analyses within the SCC were conducted to identify potential modifiers of the association between long term PM_2.5_ and PM_10_ exposure and RTL. Participants were stratified by demographic, socioeconomic, and health characteristics, including age, gender, education, marital status, occupation, family income, smoking, alcohol use, BMI, and health conditions. Interaction terms were used to assess effect modification, and three‐knot cubic splines explored non‐linear relationships between PM_2.5_ and PM_10_ exposure and RTL.

To evaluate factors influencing RTL and biological aging metrics (phenotypic and accelerated age), we implemented eXtreme Gradient Boosting (XGBoost). We also implemented a multi‐step optimization procedure for the XGBoost algorithm to ensure model robustness and minimize the risk of overfitting. First, we used a Grid Search framework combined with 5‐fold cross‐validation to identify optimal hyperparameters, including the learning rate (eta), number of trees, and maximum tree depth (Alhakeem et al. 2022). Second, the maximum tree depth was constrained to control model complexity, and the number of boosting rounds was determined through an early stopping strategy, where training was terminated if the validation error failed to improve for 10 consecutive iterations (Atias et al. 2025). Third, stochastic regularization was introduced via the subsample and colsample_bytree parameters to reduce the likelihood of the model fitting to noise and to enhance generalizability. To improve interpretability, we applied the SHAP values, which quantified the relative contribution of each feature (Hettikankanamage et al. 2025; Nohara et al. 2022).

Sensitivity analyses were conducted to assess the robustness of the findings. These included repeating the main analyses using a 2‐year average concentration of PM_2.5_ and PM_10_ to evaluate the consistency of associations with biological aging indicators. Additionally, SHAP plots were used to examine changes in the importance of air pollution exposure as predictors of biological aging metrics. Cox proportional hazards models were used to estimate adjusted hazard ratios (HRs) and 95% CIs for all‐cause mortality associated with PM_2.5_ and PM_10_ exposure, using GBCS data from 2003 to 2004 to November 21, 2023 (*N* = 9527). All statistical analyses were performed using R software (version 4.2.0). To account for multiple comparisons across the examined exposures (PM_2.5_ and PM_10_) and outcomes (phenotypic age, accelerated age, and relative telomere length), false discovery rate (FDR) correction was applied using the Benjamini–Hochberg procedure. Statistical significance was defined as a two‐sided FDR‐adjusted *p*‐value < 0.05.

## 3 Results

### 3.1 Demographic Characteristics

This study included 9527 participants, with a mean (SD) age of 64.6 (6.0) years. Of them, 29.3% were male, 50.1% had a primary education or below, 31.1% had a manual occupation, and 7% reported family income of less than 10,000 CNY/year. Additionally, 79.4% had never smoked, and 83.4% had never consumed alcohol. Participants exposed to higher levels of long‐term PM_2.5_ and PM_10_ were more likely to be older, with a higher prevalence of lower educational levels and higher physical activity, as well as a greater tendency for never smoking and never drinking (Table 1).

**TABLE 1:** Characteristics of participants in the Guangzhou Biobank Cohort Study.

Characteristics	Total (*N* = 9527)	1‐year average PM_2.5_				1‐year average PM_10_			
		Tertile 1 (*N* = 3198)	Tertile 2 (*N* = 3159)	Tertile 3 (*N* = 3170)	*p*	Tertile 1 (*N* = 3180)	Tertile 2 (*N* = 3178)	Tertile 3 (*N* = 3169)	*p*
**PDI, Median (Q1, Q3)**	49 (46, 52)	49 (46, 52)	49 (46, 52)	49 (46, 53)	0.004	49 (46, 52)	49 (46, 52)	49 (46, 53)	0.095
**PHD, Mean ± SD**	61.2 ± 11.6	60.6 ± 11.3	61.1 ± 11.7	61.9 ± 11.7	< 0.001	60.6 ± 11.4	61.2 ± 11.6	61.8 ± 11.8	< 0.001
**Temperature, °C, Median (Q1, Q3)**	22.6 (22.5, 22.7)	22.6 (22.5, 22.7)	22.6 (22.5, 22.6)	22.6 (22.5, 22.7)	< 0.001	22.6 (22.5, 22.7)	22.6 (22.5, 22.7)	22.6 (22.5, 22.7)	< 0.001
**Humidity, %, Median (Q1, Q3)**	72 (71.7, 72)	72 (71.9, 72.2)	72 (71.9, 72.2)	72 (71.4, 72)	< 0.001	72 (71.9, 72.2)	72 (71.9, 72)	72 (71.4, 72)	< 0.001
**O**_**3**_, **μg/m** ^**3**^, **Median (Q1, Q3)**	94.7 (80.7, 112)	104.9 (94.9, 115.8)	94.2 (81.8, 113.5)	79.2 (71.7, 93.4)	< 0.001	104.8 (94.9, 115.5)	92.9 (80.8, 112.4)	80.1 (71.7, 95.9)	< 0.001
**Age, years, %**					< 0.001				< 0.001
< 65	4970 (52.2)	1698 (53.1)	1751 (55.4)	1521 (48.0)		1695 (53.3)	1758 (55.3)	1517 (47.9)	
≥ 65	4557 (47.8)	1500 (46.9)	1408 (44.6)	1649 (52.0)		1485 (46.7)	1420 (44.7)	1652 (52.1)	
**Sex, %**					< 0.001				< 0.001
Men	2787 (29.3)	1012 (31.6)	968 (30.6)	807 (25.5)		1002 (31.5)	978 (30.8)	807 (25.5)	
Women	6740 (70.7)	2186 (68.4)	2191 (69.4)	2363 (74.5)		2178 (68.5)	2200 (69.2)	2362 (74.5)	
**Education level, %**					0.296				0.053
Primary or below	4776 (50.1)	1559 (48.7)	1591 (50.4)	1626 (51.3)		1554 (48.9)	1566 (49.3)	1656 (52.3)	
Secondary	3887 (40.8)	1331 (41.6)	1285 (40.6)	1271 (40.1)		1320 (41.5)	1323 (41.6)	1244 (39.3)	
College or above	864 (9.1)	308 (9.7)	283 (9.0)	273 (8.6)		306 (9.6)	289 (9.1)	269 (8.4)	
**Occupation, %**					0.174				0.005
Manual	2963 (31.1)	1025 (32.0)	969 (30.7)	969 (30.6)		1019 (32.0)	1002 (31.5)	942 (29.7)	
Non‐manual	6095 (64.0)	2014 (63.0)	2018 (63.9)	2063 (65.1)		2001 (62.9)	1996 (62.8)	2098 (66.2)	
Other	469 (4.9)	159 (5.0)	172 (5.4)	138 (4.3)		160 (5.1)	180 (5.7)	129 (4.1)	
**Family income, CNY/year, %**					0.028				0.038
< 10,000	663 (7.0)	225 (7.0)	221 (7.0)	217 (6.8)		223 (7.0)	217 (6.8)	223 (7.0)	
10,000–29,999	3205 (33.6)	1097 (34.3)	1047 (33.1)	1061 (33.5)		1081 (34.0)	1062 (33.4)	1062 (33.5)	
30,000–49,999	1452 (15.2)	525 (16.4)	480 (15.2)	447 (14.1)		518 (16.3)	488 (15.4)	446 (14.1)	
≥ 50,000	992 (10.4)	343 (10.7)	340 (10.8)	309 (9.7)		342 (10.8)	348 (11.0)	302 (9.5)	
Don't know	3215 (33.8)	1008 (31.6)	1071 (33.9)	1136 (35.9)		1016 (31.9)	1063 (33.4)	1136 (35.9)	
**Smoking status, %**					0.013				0.003
Never	7565 (79.4)	2503 (78.3)	2480 (78.5)	2582 (81.5)		2493 (78.4)	2486 (78.2)	2586 (81.6)	
Former	1010 (10.6)	360 (11.3)	342 (10.8)	308 (9.7)		354 (11.1)	344 (10.8)	312 (9.8)	
Current	952 (10.0)	335 (10.4)	337 (10.7)	280 (8.8)		333 (10.5)	348 (11.0)	271 (8.6)	
**Alcohol use, %**					0.177				0.190
Never	7950 (83.4)	2694 (84.2)	2621 (83.0)	2635 (83.1)		2683 (84.4)	2636 (82.9)	2631 (83.0)	
Former	206 (2.2)	60 (1.9)	83 (2.6)	63 (2.0)		60 (1.9)	82 (2.6)	64 (2.0)	
Current	1371 (14.4)	444 (13.9)	455 (14.4)	472 (14.9)		437 (13.7)	460 (14.5)	474 (15.0)	
**BMI, kg/m** ^**2**^, **%**					0.013				0.024
< 18.5	460 (4.8)	159 (5.0)	159 (5.0)	142 (4.5)		159 (5.0)	153 (4.8)	148 (4.7)	
18.5–24.9	5798 (60.9)	2015 (63.0)	1882 (59.6)	1901 (60.0)		2001 (62.9)	1912 (60.2)	1885 (59.5)	
25.0–27.4	2057 (21.6)	670 (21.0)	688 (21.8)	699 (22.1)		666 (20.9)	687 (21.6)	704 (22.2)	
≥ 27.5	1212 (12.7)	354 (11.0)	430 (13.6)	428 (13.4)		354 (11.2)	426 (13.4)	432 (13.6)	
**Physical activity, %**					< 0.001				< 0.001
Inactive	137 (1.4)	44 (1.4)	41 (1.3)	52 (1.6)		44 (1.4)	43 (1.3)	50 (1.6)	
Moderate	3325 (34.9)	1002 (31.3)	1081 (34.2)	1242 (39.2)		1003 (31.5)	1080 (34.0)	1242 (39.2)	
Active	6065 (63.7)	2152 (67.3)	2037 (64.5)	1876 (59.2)		2133 (67.1)	2055 (64.7)	1877 (59.2)	
**Household air pollution, %**					0.443				0.727
No	9405 (98.7)	3161 (98.8)	3112 (98.5)	3132 (98.8)		3143 (98.8)	3137 (98.7)	3125 (98.6)	
Yes	122 (1.3)	37 (1.2)	47 (1.5)	38 (1.2)		37 (1.2)	41 (1.3)	44 (1.4)	
**Passive smoking exposure, %**					< 0.001				< 0.001
< 2 years of 40 h per week	1582 (16.6)	608 (19.0)	521 (16.5)	453 (14.3)		599 (18.8)	526 (16.6)	457 (14.4)	
2–5 years of 40 h per week	1157 (12.1)	323 (10.1)	358 (11.3)	476 (15.0)		321 (10.1)	373 (11.7)	463 (14.6)	
> 5 years of 40 h per week	1953 (20.5)	597 (18.7)	623 (19.7)	733 (23.1)		601 (18.9)	625 (19.7)	727 (22.9)	
Not reported	4835 (50.8)	1670 (52.2)	1657 (52.5)	1508 (47.6)		1659 (52.2)	1654 (52.0)	1522 (48.1)	
**Arthritis, %**					< 0.001				< 0.001
No	7164 (75.2)	2301 (72.0)	2344 (74.2)	2519 (79.5)		2286 (71.9)	2338 (73.6)	2540 (80.2)	
Yes	2363 (24.8)	897 (28.0)	815 (25.8)	651 (20.5)		894 (28.1)	840 (26.4)	629 (19.8)	
**Diabetes, %**					0.313				0.800
No	8656 (90.9)	2899 (90.7)	2890 (91.5)	2867 (90.4)		2887 (90.8)	2896 (91.1)	2873 (90.7)	
Yes	871 (9.1)	299 (9.3)	269 (8.5)	303 (9.6)		293 (9.2)	282 (8.9)	296 (9.3)	
**Hypertension, %**					0.675				0.587
No	6485 (68.1)	2174 (68.0)	2168 (68.6)	2143 (67.6)		2175 (68.4)	2175 (68.4)	2135 (67.4)	
Yes	3042 (31.9)	1024 (32.0)	991 (31.4)	1027 (32.4)		1005 (31.6)	1003 (31.6)	1034 (32.6)	
**Dyslipidaemia, %**					0.341				0.344
No	8656 (90.9)	2892 (90.4)	2889 (91.5)	2875 (90.7)		2871 (90.3)	2891 (91.0)	2894 (91.3)	
Yes	871 (9.1)	306 (9.6)	270 (8.5)	295 (9.3)		309 (9.7)	287 (9.0)	275 (8.7)	
**Cardiovascular disease, %**					0.735				0.903
No	8925 (93.7)	2993 (93.6)	2968 (94.0)	2964 (93.5)		2978 (93.6)	2982 (93.8)	2965 (93.6)	
Yes	602 (6.3)	205 (6.4)	191 (6.0)	206 (6.5)		202 (6.4)	196 (6.2)	204 (6.4)	
**COPD, %**					0.774				0.444
No	7768 (81.5)	2613 (81.7)	2583 (81.8)	2572 (81.1)		2592 (81.5)	2572 (80.9)	2604 (82.2)	
Yes	1759 (18.5)	585 (18.3)	576 (18.2)	598 (18.9)		588 (18.5)	606 (19.1)	565 (17.8)	

Abbreviations: BMI, body mass index; CNY, Chinese yuan; COPD, chronic obstructive pulmonary disease; PDI, plant‐based diets index; PHD, planetary‐health diets; PM_10_, Particulate matter 10; PM_2.5_, Particulate matter 2.5; Q1, Quartile 1; Q3, Quartile 3; SD, Standard deviation.

### 3.2 Long‐Term PM Exposure and Biological Aging by PRS

Table 2 shows the associations of long‐term PM_2.5_ and PM_10_ exposure with phenotypic age and accelerated age, stratified by PRS on 1604 participants.

**TABLE 2:** Associations of long‐term exposure to 1‐year average PM_2.5_ and PM_10_ with phenotypic age (years) and accelerated age, stratified by PRS in GBCS (*N* = 1604).

1‐year average	Phenotypic age	*p*_FDR_	Accelerated age	*p*_FDR_
	*β* (95% CI)^a^		OR (95% CI)^a^	
**PM**_**2.5**_				
Per 1 μg/m^3^ increase in PM_2.5_	0.022 (−0.004, 0.048)	0.097	1.006 (0.998, 1.015)	0.153
**Stratified by PRS**				
Lower PRS				
Per 1 μg/m^3^ increase in PM_2.5_	0.040 (0.001, 0.078) *	0.048	1.013 (1.002, 1.026) *	0.045
Higher PRS				
Per 1 μg/m^3^ increase in PM_2.5_	0.008 (−0.024, 0.040)	0.626	1.002 (0.990, 1.014)	0.748
PM_10_				
Per 1 μg/m^3^ increase in PM_10_	0.014 (−0.005, 0.034)	0.154	1.004 (0.997, 1.010)	0.268
**Stratified by PRS**				
Lower PRS				
Per 1 μg/m^3^ increase in PM_10_	0.031 (0.001, 0.063) *	0.046	1.009 (1.000, 1.019)	0.054
Higher PRS				
Per 1 μg/m^3^ increase in PM_10_	0.002 (−0.021, 0.026)	0.839	1.000 (0.991, 1.009)	0.983

*Note:* Phenotypic age: P for interaction with PRS: 0.045 for PM_2.5_, 0.034 for PM_10_. Accelerated age: P for interaction with PRS: 0.033 for PM_2.5_, 0.053 for PM_10_.

Abbreviations: CI, confidence interval; OR, odds ratio; PM_10_, Particulate matter 10; PM_2.5_, Particulate matter 2.5; PRS, polygenic risk score.

^a^ Adjusted for age, sex, education level, occupation, family income, smoking status, alcohol use, BMI, physical activity, household air pollution, passive smoking exposure, temperature, humidity, O_3_, arthritis, diabetes, hypertension, dyslipidaemia, cardiovascular disease, COPD.

*p_FDR_ < 0.05; **p_FDR_ < 0.01; ***p_FDR_ < 0.001.

Interaction analyses revealed significant PM × PRS interactions in relation to phenotypic age (*β*_PM2.5_ = −0.051 years, *p* = 0.045; *β*_PM10_ = −0.041 years, *p* = 0.034) and a significant PM_10_ × PRS interaction associated with accelerated aging (OR = 0.989, *p* = 0.033). PM_2.5_ × PRS was not significant for accelerated aging (OR = 0.987, *p* = 0.053) (Table 2). Each 1 μg/m^3^ increase in PM was associated with a non‐significant increase in phenotypic age (*β*_PM2.5_ = 0.022 years; 95% CI: −0.004, 0.048, *β*_PM10_ = 0.014 years; −0.005, 0.034), and increased odds of accelerated aging (OR_PM2.5_ = 1.006; 95% CI: 0.998, 1.015, OR_PM10_ = 1.004; 95% CI: 0.997, 1.010). These associations were more pronounced in participants with lower PRS compared to those with higher PRS.

### 3.3 Associations of Long‐Term PM_2.5_ and PM_10_ Exposure With RTL in the SCC

**FIGURE 1:**
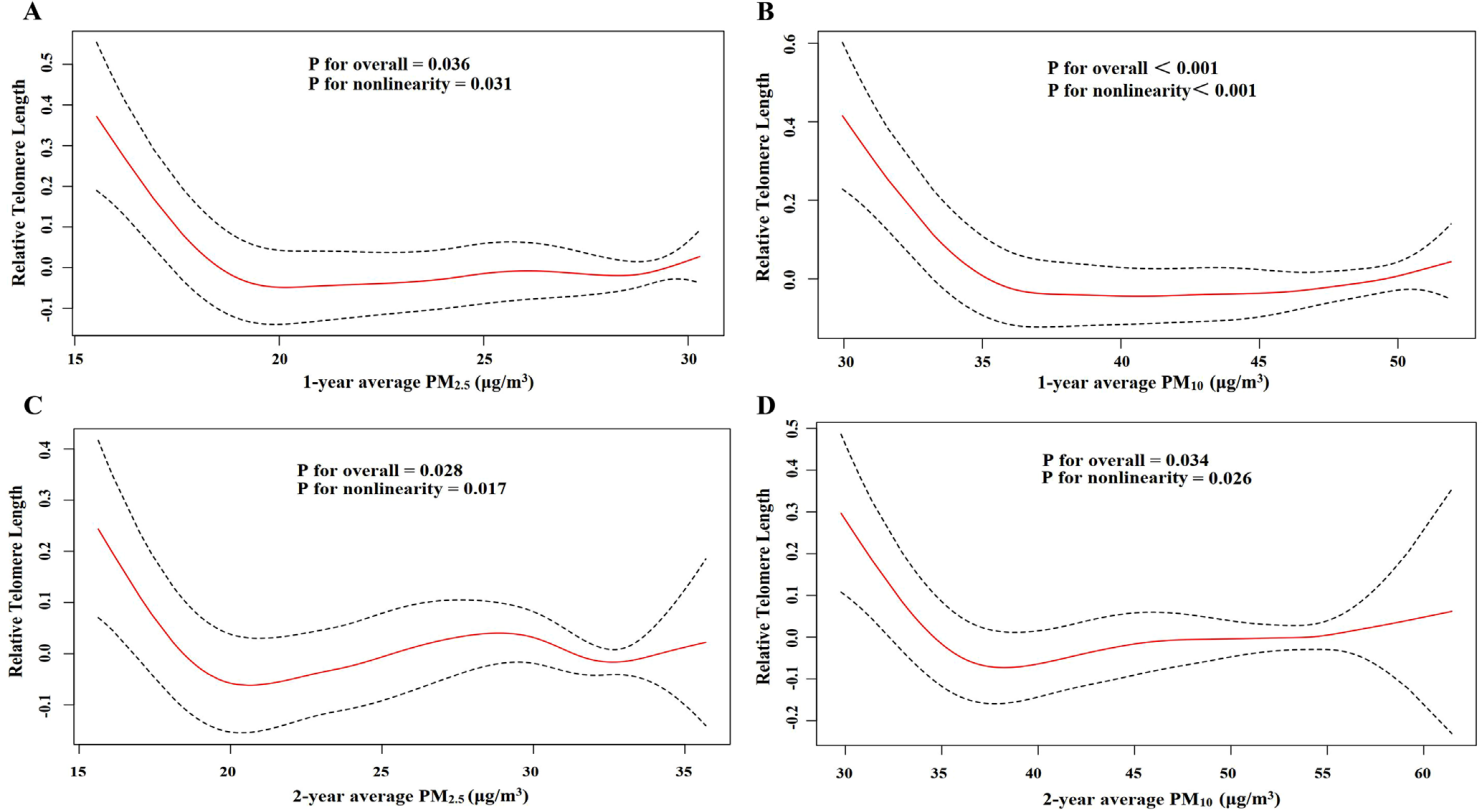
Associations of 1‐year and 2‐year average PM_2.5_ and PM_10_ concentrations (μg/m^3^) with relative telomere length in the SCC. Solid red lines represent the estimated relative telomere length, and dashed black lines indicate 95% confidence intervals (CIs). Panels: (A) 1‐year PM_2.5_; (B) 1‐year PM_10_; (C) 2‐year PM_2.5_; and (D) 2‐year PM_10_. Models adjusted for age, sex, education level, occupation, family income, smoking status, alcohol use, BMI, physical activity, household air pollution, passive smoking exposure, temperature, humidity, NO_2_, O_3_, arthritis, diabetes, hypertension, dyslipidaemia, cardiovascular disease. BMI, body mass index; CI, confidence interval; NO_2_, nitrogen dioxide; O_3_, ozone; PM, particulate matter; SCC, South China Cohort.

Table 3 shows a potential trend in the association of PM_2.5_ and PM_10_ exposure levels with RTL shortening, although these associations did not reach statistical significance (*β*_PM2.5_ = −0.005; 95% CI: −0.012, 0.003, *β*_PM10_ = −0.001; −0.006, 0.005). Specifically, age‐stratified analyses indicate that the negative association between PM exposure and RTL was more pronounced in older individuals (≥ 65 years). No significant associations were observed for younger participants. The association also appeared stronger among non‐smokers, those with cardiovascular disease, and those with dyslipidemia. Notably, nonlinear patterns were observed in Figure 1, with more pronounced reductions in RTL at moderate pollution levels, followed by an attenuation at higher levels.

**TABLE 3:** Associations of long‐term exposure to 1‐year average PM_2.5_ and PM_10_ with relative telomere length (RTL), stratified by demographic variables in South China Cohort (SCC) (*N* = 2023).

	1‐year average PM_2.5_			1‐year average PM_10_		
	*β* (95% CI)	*p*_FDR_	*p*‐value for interaction	*β* (95% CI)	*p*_FDR_	*p*‐value for interaction
**All participants**	−0.005 (−0.012, 0.003)	0.217	—	−0.001 (−0.006, 0.005)	0.778	—
**Age, years**			0.111			0.164
< 65	−0.002 (−0.011, 0.006)	0.584		0.001 (−0.005, 0.006)	0.802	
≥ 65	−0.020 (−0.039, 0.001) *	0.048		−0.010 (−0.023, 0.004)	0.155	
**Sex**			0.820			0.736
Men	−0.003 (−0.017, 0.010)	0.623		0.001 (−0.009, 0.010)	0.894	
Women	−0.005 (−0.015, 0.004)	0.254		−0.001 (−0.008, 0.005)	0.679	
**Education level**			0.166			0.095
Primary or below	0.025 (−0.008, 0.058)	0.138		0.022 (−0.001, 0.045)	0.059	
Secondary	−0.007 (−0.015, 0.002)	0.131		−0.003 (−0.009, 0.003)	0.339	
College or above	−0.008 (−0.029, 0.013)	0.463		0.002 (−0.012, 0.016)	0.798	
**Marital status**			0.251			0.327
Never married	0.072 (−0.006, 0.15)	0.092		0.048 (−0.008, 0.104)	0.115	
Married	−0.005 (−0.013, 0.003)	0.185		−0.001 (−0.006, 0.004)	0.712	
Separated/divorced/widowed	−0.007 (−0.041, 0.028)	0.710		−0.003 (−0.027, 0.022)	0.833	
**Occupation**			0.412			0.279
Manual	−0.011 (−0.025, 0.002)	0.100		−0.006 (−0.016, 0.003)	0.179	
Non‐manual	0.001 (−0.012, 0.014)	0.858		0.002 (−0.007, 0.012)	0.612	
Other	−0.002 (−0.016, 0.011)	0.735		0.003 (−0.006, 0.012)	0.492	
**Family income, CNY/year**			0.982			0.424
< 50,000	−0.003 (−0.019, 0.013)	0.722		−0.001 (−0.012, 0.01)	0.263	
50,000–79,999	−0.001 (−0.015, 0.014)	0.928		0.004 (−0.006, 0.014)	0.413	
≥ 80,000	−0.004 (−0.015, 0.007)	0.445		−0.001 (−0.008, 0.007)	0.593	
Don't know	−0.007 (−0.058, 0.045)	0.807		−0.007 (−0.044, 0.03)	0.538	
**Smoking status**			0.072			0.079
Never	−0.009 (−0.017, −0.001) *	0.042		−0.003 (−0.009, 0.002)	0.244	
Former	0.013 (−0.013, 0.039)	0.343		0.014 (−0.004, 0.032)	0.130	
Current	0.016 (−0.009, 0.04)	0.209		0.011 (−0.006, 0.028)	0.209	
**Alcohol use**			0.945			0.994
Never	−0.005 (−0.013, 0.004)	0.264		−0.001 (−0.006, 0.005)	0.858	
Former	−0.001 (−0.021, 0.019)	0.913		0.001 (−0.013, 0.014)	0.970	
Current	−0.005 (−0.046, 0.036)	0.817		−0.001 (−0.029, 0.028)	0.963	
**BMI, kg/m** ^**2**^			0.699			0.693
< 18.5	0.002 (−0.044, 0.049)	0.917		0.009 (−0.023, 0.042)	0.576	
18.5–24.9	−0.005 (−0.014, 0.004)	0.267		−0.001 (−0.007, 0.006)	0.849	
25.0–27.4	0.001 (−0.017, 0.018)	0.974		0.001 (−0.011, 0.013)	0.920	
≥ 27.5	−0.019 (−0.047, 0.009)	0.177		−0.010 (−0.029, 0.008)	0.286	
**Arthritis**			0.264			0.177
No	−0.004 (−0.011, 0.004)	0.385		0.001 (−0.005, 0.006)	0.910	
Yes	−0.020 (−0.047, 0.008)	0.160		−0.013 (−0.032, 0.006)	0.174	
**Diabetes**			0.189			0.377
No	−0.003 (−0.012, 0.005)	0.471		0.001 (−0.006, 0.006)	0.977	
Yes	−0.017 (−0.033, 0.001)	0.052		−0.006 (−0.018, 0.005)	0.285	
**Hypertension**			0.493			0.367
No	−0.003 (−0.012, 0.006)	0.485		0.001 (−0.006, 0.007)	0.831	
Yes	−0.009 (−0.023, 0.005)	0.189		−0.005 (−0.014, 0.005)	0.325	
**Dyslipidaemia**			0.046			0.065
No	−0.001 (−0.009, 0.01)	0.919		0.003 (−0.004, 0.009)	0.406	
Yes	−0.016 (−0.030, −0.002) *	0.024		−0.008 (−0.018, 0.002)	0.106	
**Cardiovascular disease**			0.003			0.078
No	−0.005 (−0.012, 0.003)	0.243		−0.001 (−0.006, 0.005)	0.817	
Yes	−0.487 (−0.678, −0.295) ***	< 0.001		−0.152 (−0.311, 0.008)	0.090	

Abbreviations: BMI, body mass index; CNY, Chinese yuan; PM_10_, Particulate matter 10; PM_2.5_, Particulate matter 2.5.

*p_FDR_ < 0.05; **p_FDR_ < 0.01; ***p_FDR_ < 0.001.

### 3.4 Modifying Role of Sustainable Diets

**FIGURE 2:**
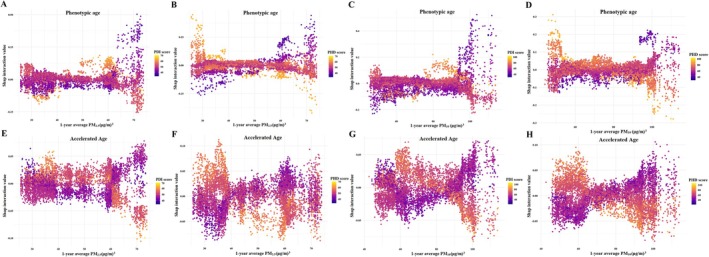
SHAP interaction plots for 1‐year average PM_2.5_ and PM_10_ exposure (μg/m^3^) and individual PDI/PHD scores in relation to phenotypic age (years) and accelerated age in the GBCS. The color gradient represents the value of the interaction feature (PDI or PHD score), ranging from low (purple) to high (yellow). Panels display interactions for Phenotypic Age (A–D) and Accelerated Age (E–H): (A, E) PM_2.5_ and PDI scores; (B, F) PM_2.5_ and PHD scores; (C, G) PM_10_ and PDI scores; (D, H) PM_10_ and PHD scores. Models adjusted for age, sex, education level, occupation, family income, smoking status, alcohol use, BMI, physical activity, household air pollution, passive smoking exposure, temperature, humidity, O_3_, arthritis, diabetes, hypertension, dyslipidaemia, cardiovascular disease, COPD. BMI, body mass index; COPD, chronic obstructive pulmonary disease; GBCS, Guangzhou Biobank Cohort Study; O_3_, ozone; PDI, Plant‐Based Diet Index; PHD, Planetary Health Diet; PM, particulate matter; SHAP, SHapley Additive exPlanations.

**FIGURE 3:**
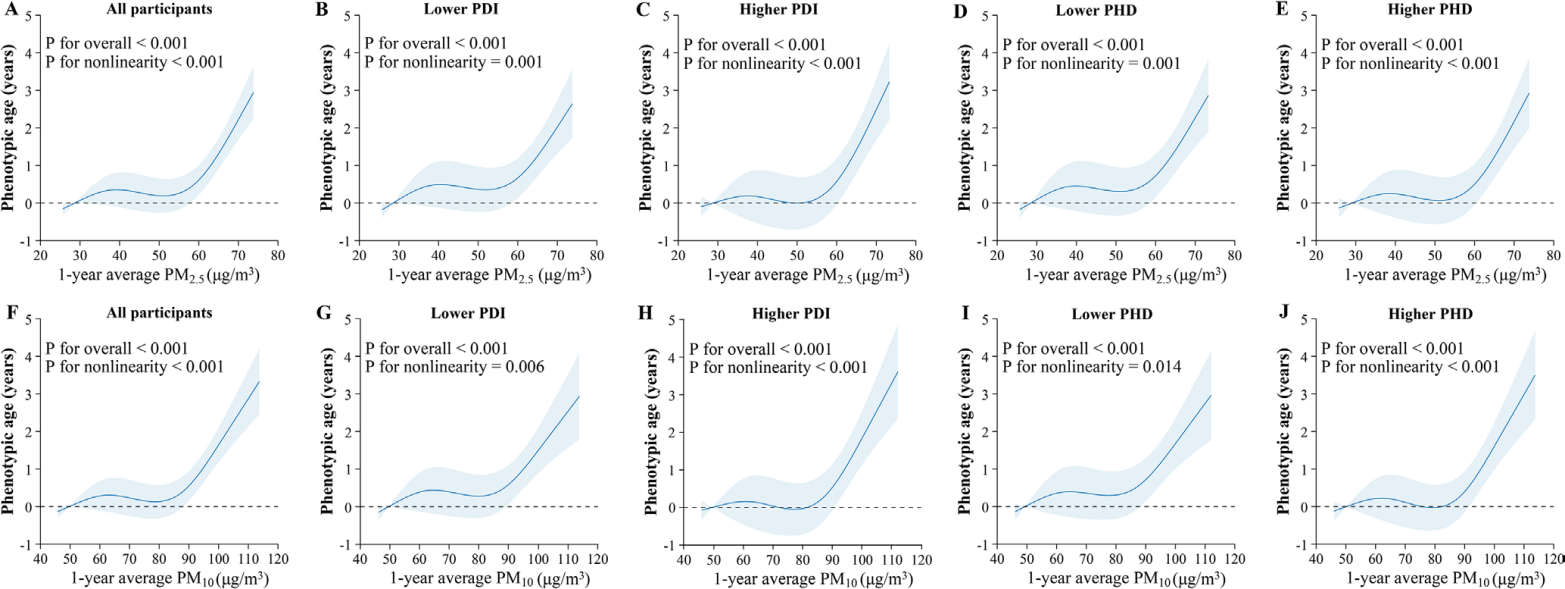
Associations of 1‐year average PM_2.5_ and PM_10_ concentrations (μg/m^3^) with phenotypic age (years), stratified by PDI and PHD in the GBCS. Panels A–E: PM_2.5_, and panels F–J: PM_10_. The first panels (A, F) show the total sample, others show stratification by diet adherence. Solid lines represent *β* coefficients, and shaded areas indicate 95% confidence intervals (CIs). Models adjusted for age, sex, education level, occupation, family income, smoking status, alcohol use, BMI, physical activity, household air pollution, passive smoking exposure, temperature, humidity, O_3_, arthritis, diabetes, hypertension, dyslipidaemia, cardiovascular disease, COPD. BMI, body mass index; CI, confidence interval; COPD, chronic obstructive pulmonary disease; GBCS, Guangzhou Biobank Cohort Study; O_3_, ozone; PDI, Plant‐Based Diet Index; PHD, Planetary Health Diet; PM, particulate matter.

**FIGURE 4:**
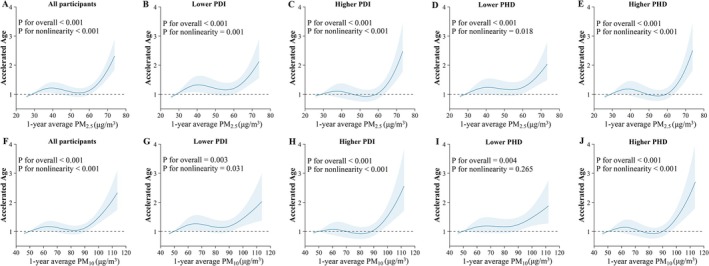
Associations of 1‐year average PM_2.5_ and PM_10_ concentrations (μg/m^3^) with accelerated age, stratified by PDI and PHD in the GBCS. Panels A–E: PM_2.5_, and panels F–J: PM_10_. Panels A and F represent the total sample, others show stratification by diet adherence. Solid lines represent odds ratios (ORs), and shaded areas indicate 95% confidence intervals (CIs). Models adjusted for age, sex, education level, occupation, family income, smoking status, alcohol use, BMI, physical activity, household air pollution, passive smoking exposure, temperature, humidity, O_3_, arthritis, diabetes, hypertension, dyslipidaemia, cardiovascular disease, COPD. BMI, body mass index; CI, confidence interval; COPD, chronic obstructive pulmonary disease; GBCS, Guangzhou Biobank Cohort Study; O_3_, ozone; OR, odds ratio; PDI, Plant‐Based Diet Index; PHD, Planetary Health Diet; PM, particulate matter.

Figure 2 presents SHAP interaction plots illustrating the interactions between long‐term PM_2.5_ or PM_10_ exposure and dietary indices (PDI and PHD) in relation to phenotypic age and accelerated aging. Higher adherence to PDI and PHD attenuated the associations between air pollution and biological aging. Specifically, among participants with higher PDI and PHD scores, the detrimental associations between elevated PM_2.5_ or PM_10_ concentrations and biological aging metrics were no longer statistically significant. Table 4 shows that, in both crude and adjusted models, increases in PM_2.5_ and PM_10_ concentrations were significantly associated with older phenotypic age (*β*_PM2.5_ = 0.039 years; 95% CI: 0.028, 0.050, *β*_PM10_ = 0.028 years; 0.020, 0.035) and the risk of accelerated age (OR_PM2.5_ = 1.008; 95% CI: 1.004, 1.012, OR_PM10_ = 1.005; 1.003, 1.008). The associations were weaker among those with higher PDI and PHD scores. The RCS analysis results show a significant increase in phenotypic age and accelerated age with higher PM_2.5_ and PM_10_ exposure, particularly at high pollution levels. However, this association appears weaker in groups with higher PDI and PHD scores (Figures 3 and 4).

**TABLE 4:** Associations of long‐term exposure to 1‐year average PM_2.5_ and PM_10_ with phenotypic age (years) and accelerated age, stratified by Plant‐Based and Planetary Health Diets in GBCS (*N* = 9527).

1‐year average	Phenotypic age, *β* (95% CI)				Accelerated age, OR (95% CI)			
	Crude model	*p*_FDR_	Adjusted model^a^	*p*_FDR_	Crude model	*p*_FDR_	Adjusted model^a^	*p*_FDR_
**PM**_**2.5**_								
Per 1 μg/m^3^ increase in PM_2.5_	0.047 (0.034, 0.061) ***	< 0.001	0.039 (0.028, 0.050) ***	< 0.001	1.005 (1.002, 1.008) **	0.004	1.008 (1.004, 1.012) ***	< 0.001
**Stratified by PDI**								
Lower PDI								
Per 1 μg/m^3^ increase in PM_2.5_	0.070 (0.050, 0.091) ***	< 0.001	0.048 (0.032, 0.065) ***	< 0.001	1.008 (1.004, 1.013) **	0.003	1.009 (1.004, 1.014) **	0.003
Higher PDI								
Per 1 μg/m^3^ increase in PM_2.5_	0.028 (0.011, 0.046) **	0.002	0.033 (0.019, 0.046) ***	< 0.001	1.002 (1, 1.006) *	0.033	1.007 (1.003, 1.012) **	0.004
**Stratified by PHD**								
Lower PHD								
Per 1 μg/m^3^ increase in PM_2.5_	0.050 (0.031, 0.069) ***	< 0.001	0.040 (0.026, 0.055) ***	< 0.001	1.0052 (1.001, 1.009) *	0.029	1.0082 (1.0032, 1.013) **	0.003
Higher PHD								
Per 1 μg/m^3^ increase in PM_2.5_	0.047 (0.028, 0.066) ***	< 0.001	0.038 (0.023, 0.053) ***	< 0.001	1.0046 (1.000, 1.009) *	0.044	1.0079 (1.003, 1.013) **	0.004
PM_10_								
Per 1 μg/m^3^ increase in PM_10_	0.037 (0.026, 0.047) ***	< 0.001	0.028 (0.020, 0.035) ***	< 0.001	1.003 (1.001, 1.006) **	0.008	1.005 (1.003, 1.008) ***	< 0.001
**Stratified by PDI**								
Lower PDI								
Per 1 μg/m^3^ increase in PM_10_	0.054 (0.039, 0.070) ***	< 0.001	0.034 (0.022, 0.047) ***	< 0.001	1.006 (1.003, 1.009) **	0.003	1.006 (1.002, 1.010) **	0.004
Higher PDI								
Per 1 μg/m^3^ increase in PM_10_	0.022 (0.009, 0.036) **	0.002	0.023 (0.013, 0.033) ***	< 0.001	1.001 (1.000, 1.004) *	0.041	1.0047 (1.001, 1.008) **	0.008
**Stratified by PHD**								
Lower PHD								
Per 1 μg/m^3^ increase in PM_10_	0.038 (0.024, 0.053) ***	< 0.001	0.029 (0.018, 0.040) ***	< 0.001	1.004 (1.001, 1.007) *	0.038	1.0054 (1.002, 1.009) **	0.006
Higher PHD								
Per 1 μg/m^3^ increase in PM_10_	0.037 (0.022, 0.051) ***	< 0.001	0.027 (0.015, 0.038) ***	< 0.001	1.0032 (1.000, 1.006) *	0.046	1.0043 (1.001, 1.009) *	0.006

Abbreviations: CI, confidence interval; OR, odds ratio; PDI, plant‐based diets index; PHD, planetary‐health diets; PM_10_, Particulate matter 10; PM_2.5_, Particulate matter 2.5.

^a^ Adjusted for age, sex, education level, occupation, family income, smoking status, alcohol use, BMI, physical activity, household air pollution, passive smoking exposure, temperature, humidity, O_3_, arthritis, diabetes, hypertension, dyslipidaemia, cardiovascular disease, COPD.

*p_FDR_ < 0.05; **p_FDR_ < 0.01; ***p_FDR_ < 0.001.

### 3.5 Sensitivity Analysis

First, sensitivity analyses using 2‐year average PM_2.5_ and PM_10_ concentrations yielded consistent results, showing significant associations with older phenotypic age and higher odds of accelerated aging. These associations were more pronounced among individuals with lower PDI and PHD scores (Tables S2–S4 and S6–S10; Figures S5–S7). Second, 1‐year and 2‐year average PM_2.5_ and PM_10_ exposures were associated with higher risks of all‐cause mortality in the GBCS from 2003 to 2004 to 2023 (Table S5).

Third, SHAP analyses indicated that the SHAP values for phenotypic age and accelerated age exhibited a progressive increase associated with higher PM_2.5_ and PM_10_ exposure levels (Figures S8–S11). Fourth, Figure S12 shows the relative importance of predictors for relative telomere length in the SCC, while Figure S13 presents the relative importance of predictors for phenotypic age and accelerated age in the GBCS. Fifth, sensitivity analyses applying a more stringent accelerated aging cutoff of 5 years (Lu et al. 2025) produced consistent results, with PM_2.5_ and PM_10_ concentrations remaining positively associated with accelerated aging, particularly among participants with lower PDI and PHD scores (Table S11). Finally, baseline demographic, socioeconomic, lifestyle, and clinical characteristics across tertiles of PM_2.5_ and PM_10_ exposure of the SCC sample are summarized in Table S12.

## 4 Discussion

This study shows significant associations of long‐term exposure to PM_2.5_ and PM_10_ with biological aging, as evidenced by increased phenotypic age, accelerated aging metrics, and shorter telomere length. Notably, our findings indicate that adherence to sustainable diets (PDI and PHD) attenuated the positive association of PM exposure with biological aging. Furthermore, individuals with lower PRS for longevity appeared more vulnerable to the associations between PM exposure and accelerated biological aging. This vulnerability highlights an important gene–environment interaction. Additionally, the association between PM exposure and shortened RTL was stronger in older adults, non‐smokers, and those with pre‐existing cardiovascular conditions, underscoring the potential of sustainable diets intervention for vulnerable subpopulations.

Our results are consistent with previous studies showing a positive association between long‐term exposure to PM_2.5_ and PM_10_ and accelerated aging in adults (Liang et al. 2024; Pan et al. 2023; Ward‐Caviness et al. 2016). Long‐term PM_2.5_ exposure has been associated with higher risks of cardiovascular and musculoskeletal disorders (Guo et al. 2024; Wang et al. 2024), potentially through inflammatory pathways, oxidative stress and epigenetic modification (Pan et al. 2023; Park et al. 2024; Puckett et al. 2024; White et al. 2019). Similarly, PM_10_ exposure, although characterized by larger particle size and limited pulmonary penetration compared to PM_2.5_, contributes to systematic inflammation and oxidative damage (Pu et al. 2024; Ullah et al. 2021; Zheng et al. 2023). Our findings also align with studies reporting significant telomere shortening associated with air pollution, a hallmark of cellular aging. Mechanistically, PM‐induced biological aging involves multiple pathways. First, PM_2.5_ induces DNA damage through oxidative mechanisms and inflammation responses triggered by reactive oxygen species (ROS) (Abbas et al. 2016; Meng and Zhang 2007; Prahalad et al. 2001). PM_2.5_ also induces systematic inflammation, alter epigenetic regulation, and disrupts mitochondrial function, thereby biological aging processes (Chen et al. 2015; Nwanaji‐Enwerem et al. 2017; Wang et al. 2017). Our observation of shorter RTL in association with higher PM exposure aligns with studies showing that each increment in annual PM exposure significantly reduced telomere length (Pieters et al. 2016; Wu et al. 2023). We found a stronger link between PM exposure and shortened RTL in older adults, likely due to reduced vascular elasticity and weakened oxidative stress defenses, which make inflammation more likely to cause cellular damage and accelerate telomere shortening (Sacks et al. 2011). Additionally, declines in telomerase activity with age reduce repair capacity, increasing the likelihood of permanent telomere shortening (Armanios et al. 2009; Yang et al. 2017). Older adults have longer duration of air pollution exposure and slower pollutant clearance, which lead to longer retention times (Liu et al. 2024; Simoni et al. 2015). Although the use of retrospective pollution data (e.g., 1‐year average) provides a temporal framework, the cross‐sectional design limits definitive causal inference. Reverse causality cannot be ruled out, that is, those with poorer health or accelerated aging may change their dietary habits, introducing bias. While we adjusted for multiple potential confounders and comorbidities, residual confounding and measurement error remain possible. Future longitudinal analyses with repeated measures of diet and aging biomarkers are warranted to validate the temporality and directionality of associations observed.

Importantly, our findings support the hypothesis that sustainable dietary patterns may offer protective effects against air pollution‐induced biological aging. These diets are rich in bioactive compounds including polyphenols, flavonoids, carotenoids and other antioxidants which have been shown to counteract ROS generated by PM exposure (Nunes et al. 2018; Serafini and Peluso 2016). Mechanistically, dietary polyphenols exert antioxidant effects through several pathways. A key mechanism involves activation of the nuclear factor erythroid 2‐related factor 2 (Nrf2) pathway. Polyphenols can modify cysteine residues on Keap1, leading to Nrf2 stabilization and nuclear translocation, where it binds to antioxidant response element (ARE) and induces expression of cytoprotective genes (Qin et al. 2023). This process enhances cellular resistance to oxidative insults, including those induced by PM_2.5_. Additionally, polyphenols inhibit the nuclear factor‐kappa B (NF‐κB) pathway, an essential regulator of pro‐inflammatory cytokine production, through epigenetic modifications and direct molecular interactions (Guan et al. 2021). Beyond molecular signaling, polyphenols directly scavenge ROS, chelate transition metal ions, and enhance endogenous antioxidant enzyme activity, thereby reducing oxidative damage to lipids, proteins, and DNA (Das et al. 2024; Guan et al. 2021). Consistent with this, higher intake of healthy plant‐based foods has been associated with lower levels of oxidative stress markers (e.g., malondialdehyde, 8‐hydroxy‐2′‐deoxyguanosine) and inflammatory cytokines (e.g., TNF‐α, IL‐1β) (Aleksandrova et al. 2021; Ilari et al. 2025). Epidemiological evidence further supports these mechanistic pathways. Adherence to plant‐based or Mediterranean diets was associated with slower cognitive decline in populations with long‐term PM exposure (Wu et al. 2019; Zhu et al. 2022) and decelerated biological aging trajectories in large cohort studies (Wang et al. 2023). Findings from the Moli‐sani cohort showed that adherence to both Mediterranean and DASH diet, characterized by high polyphenol content, was significantly associated with lower biological age (Esposito et al. 2022, 2021). Mediation analyses from these studies showed that dietary polyphenol intake explained 29.8% and 65.8% of the observed anti‐aging associations, respectively (Esposito et al. 2022).

Our identification of a higher susceptibility to PM_2.5_ and PM_10_ in those with a lower genetic predisposition for longevity emphasizes the importance of integrating genetic context into environmental health research. This observation aligns with studies demonstrating gene–environment interaction, such as those showing significantly higher psoriasis risk among individuals with high PRS when exposed to PM_2.5_ and PM_10_ (Wu et al. 2024), and more pronounced reductions in brain gray matter volume in genetically susceptible individuals upon PM exposure (Essers et al. 2023; Jung et al. 2022). Longevity‐associated genes may modulate host responses to environmental insults through their influence on oxidative stress defense, inflammatory regulation and DNA repair pathways (Pieters et al. 2016; Tang et al. 2024; Yao et al. 2021). For example, variants in the SIRT1 gene (a key regulator of cellular aging) have been associated with altered susceptibility to pollution‐related mortality. In a study, participants homozygous for the minor allele of SIRT1_391 showed significantly higher mortality risk associated with PM_2.5_ exposure (Yao et al. 2021). Mechanistically, SIRT1 activation promotes antioxidant defenses by upregulating FOXO transcription factors and suppressing NF‐κB‐mediated inflammatory pathways (Gupta et al. 2025; Hori et al. 2013). However, reactive oxygen species (ROS) generated by air pollution can oxidatively modify SIRT1, inhibiting its activity and compromising its protective effects (Salminen et al. 2013). Individuals with genetically unfavorable variants in such pathways may thus exhibit impaired antioxidant and anti‐inflammatory responses, greater telomere attrition, and higher biological vulnerability to chronic pollutant exposure (Abdulkhaliq et al. 2025; Liu et al. 2025; Yan et al. 2024).

Our study has several strengths, including the novel examination of how sustainable diets may be associated with slower biological aging in the context of air pollution exposure, the integration of extensive cohort data, and the application of advanced analytical methods such as XGBoost and SHAP. By incorporating two independent cohorts from different time periods with varying pollution levels, we were able to assess the consistency of associations across distinct exposure contexts, enhancing the generalizability and temporal relevance of our findings. Additionally, sensitivity analyses strengthen the robustness of our findings. However, several limitations should be acknowledged. First, the cross‐sectional nature of the data limits causal inference and restricts our ability to determine the temporal direction of the associations. This highlights the need for further experimental studies using animal models to confirm the modification effect of sustainable diets. Second, dietary data was assessed using a validated FFQ, which is subject to inherent limitations such as recall error and misclassification. However, such errors are likely non‐differential with respect to biological aging outcomes, which would bias associations toward the null. As such the observed effect modification by diet may be conservative. Additionally, although we adjusted for a wide range of potential confounders, residual confounding cannot be entirely ruled out. Nonetheless, the use of validated instruments and consistent results across sensitivity analyses support the robustness of the observed associations. Fourth, reliance on self‐reported fuel use may limit the ability to capture fuel stacking and the spatiotemporal variability of exposure. Future studies should consider incorporating personal exposure monitoring or biomarker‐based assessments to enhance exposure precision. Fifth, due to the lack of genetic data in the SCC cohort, a telomere length PRS could not be calculated. Future cohort studies with genotyped data are needed to validate the predictive utility of PRS for aging. Finally, the PRS model was developed based on an Asian population. Generalizability of our findings to non‐Asian populations may be limited.

## 5 Conclusion

In conclusion, our findings emphasize the detrimental associations between PM_2.5_ and PM_10_ exposure and biological aging, alongside the significant modifying role of health‐focused (PDI) and sustainability‐designed (PHD) dietary patterns. Future public health interventions could benefit from promoting sustainable diets, particularly among genetically susceptible and older populations, to mitigate the adverse health impacts of air pollution.

## Author Contributions

R.Q.L., K.K.C., and L.X. conceived and designed the study. R.Q.L., S.X.P., R.H.Z., T.Y.L., and J.W. performed experiments and data collection. R.Q.L., Y.W., and J.W. analyzed the data. L.X. and R.Q.L. wrote the original draft. W.S.Z., L.Y., S.L.R.A.Y., T.H.L., K.K.C., and L.X. supervised the project and reviewed the manuscript. L.X., T.H.L., and W.S.Z. acquired funding. All authors read and approved the final version.

## Funding

The National Natural Science Foundation of China (82373661) and Natural Science Foundation of Guangdong (2022A1515011546). The Guangzhou biobank cohort study was funded by The University of Hong Kong Foundation for Educational Development and Research (SN/1f/HKUF‐DC; C20400.28505200), the Health Medical Research Fund (Grant number: HMRF/13143241) in Hong Kong, and the University of Birmingham, UK.

## Ethics Statement

This study protocol of GBCS was reviewed and approved by the Guangzhou Medical Ethics Committee of the Chinese Medical Association and the Medical Ethics Committee of the Guangzhou Twelfth People's Hospital. SCC was approved by the Ethics Committee of School of Public Health, Sun Yat‐Sen University (L2017‐001).

## Conflicts of Interest

The authors declare no conflicts of interest.

## Supporting information


**Data S1:** acel70422‐sup‐0001‐supinfo.docx.

## Data Availability

Ethical approval in place allows us to share data on requests. Please directly send such requests to the Guangzhou Biobank Cohort Study Data Access Committee (gbcsdata@hku.hk). The South China Cohort encourages global collaboration. While sensitive data are not publicly available, researchers may contact Prof. Min Xia (xiamin@mail.sysu.edu.cn) for access proposals.
