# Supplementary material for: Modifying Role of Sustainable Diets on the Association Between Particulate Matter and Biological Aging: The Guangzhou Biobank Cohort Study
Source: Aging Cell. 2026 Feb 27;25(3):e70422. doi: 10.1111/acel.70422 (PMC12947253; doi:10.1111/acel.70422)
Supplement: Supplementary file 1 — Data S1: acel70422‐sup‐0001‐supinfo.docx. [file ACEL-25-e70422-s001.docx]

**Supplementary table 1.** List of longevity-associated SNPs used for polygenic risk score construction in the Guangzhou Biobank Cohort Study.

| **SNP** | **CHR** | **Position** | **Effect allele** | **Other allele** | **Beta** | **SE** | **P** |
| --- | --- | --- | --- | --- | --- | --- | --- |
| rs16981095 | 19 | 16190863 | G | T | 0.2109 | 0.0431 | 9.75E-07 |
| rs3803304 | 14 | 105239146 | G | C | 0.2045 | 0.0469 | 1.30E-05 |
| rs1043943 | 3 | 14183410 | T | C | 0.1464 | 0.0288 | 3.59E-08 |
| rs2075650 | 19 | 45395619 | G | A | -0.2934 | 0.0474 | 6.17E-10 |
| rs11925757 | 3 | 14241981 | G | A | 0.1324 | 0.0283 | 2.91E-06 |

SNP: single-nucleotide polymorphisms; CHR: chromosome; SE: standard error.

| **Supplementary table 2.** Associations of long-term exposure to 2-year average PM_2.5_ and PM_10_ with phenotypic age (years) and accelerated age, stratified by PDI and PHD in GBCS (N=9527) | | | | | |
| --- | --- | --- | --- | --- | --- |
| **2-year average** | **Phenotypic age, β (95 % CI)** | |  | **Accelerated age, OR (95 % CI)** | |
|  | Crude model | Adjusted model ^a^ |  | Crude model | Adjusted model ^a^ |
| **PM_2.5_** |  |  |  |  |  |
| Per 1 μg/m^3^ increase in PM_2.5_ | 0.044 (0.030, 0.058) *** | 0.033 (0.023, 0.043) *** |  | 1.005 (1.002, 1.008) ** | 1.0074 (1.004, 1.011) *** |
| **Stratified by PDI** |  |  |  |  |  |
| **Lower PDI** |  |  |  |  |  |
| Per 1 μg/m^3^ increase in PM_2.5_ | 0.066 (0.045, 0.087) *** | 0.038 (0.023, 0.054) *** |  | 1.009 (1.004, 1.013) *** | 1.009 (1.004, 1.014) *** |
| **Higher PDI** |  |  |  |  |  |
| Per 1 μg/m^3^ increase in PM_2.5_ | 0.026 (0.008, 0.044) ** | 0.029 (0.016, 0.042) *** |  | 1.001 (1.000, 1.005) * | 1.006 (1.002, 1.011) ** |
| **Stratified by PHD** |  |  |  |  |  |
| **Lower PHD** |  |  |  |  |  |
| Per 1 μg/m^3^ increase in PM_2.5_ | 0.044 (0.025, 0.064) *** | 0.036 (0.021, 0.050) *** |  | 1.006 (1.001, 1.010) ** | 1.009 (1.005, 1.014) *** |
| **Higher PHD** |  |  |  |  |  |
| Per 1 μg/m^3^ increase in PM_2.5_ | 0.046 (0.027, 0.065) *** | 0.029 (0.016, 0.043) *** |  | 1.004 (1.000, 1.008) * | 1.005 (1.000, 1.010) * |
| **PM_10_** |  |  |  |  |  |
| Per 1 μg/m^3^ increase in PM_10_ | 0.034 (0.024, 0.045) *** | 0.024 (0.017, 0.032) *** |  | 1.003 (1.001, 1.006) ** | 1.0052 (1.003, 1.008) *** |
| **Stratified by PDI** |  |  |  |  |  |
| **Lower PDI** |  |  |  |  |  |
| Per 1 μg/m^3^ increase in PM_10_ | 0.051 (0.036, 0.067) *** | 0.029 (0.017, 0.040) *** |  | 1.006 (1.003, 1.010) *** | 1.006 (1.002, 1.010) ** |
| **Higher PDI** |  |  |  |  |  |
| Per 1 μg/m^3^ increase in PM_10_ | 0.020 (0.006, 0.034) ** | 0.021 (0.011, 0.031) *** |  | 1.001 (1, 1.004) * | 1.004 (1.001, 1.008) * |
| **Stratified by PHD** |  |  |  |  |  |
| **Lower PHD** |  |  |  |  |  |
| Per 1 μg/m^3^ increase in PM_10_ | 0.035 (0.020, 0.050) *** | 0.026 (0.015, 0.037) *** |  | 1.004 (1.001, 1.007) * | 1.007 (1.003, 1.010) *** |
| **Higher PHD** |  |  |  |  |  |
| Per 1 μg/m^3^ increase in PM_10_ | 0.035 (0.021, 0.050) *** | 0.022 (0.011, 0.032) *** |  | 1.002 (1.000, 1.006) * | 1.004 (1.000, 1.007) * |
| PM_2.5_: Particulate matter 2.5; PM_10_: Particulate matter 10; PDI: plant-based diets index; PHD: planetary-health diets; OR: odds ratio; CI: confidence interval.  ^a^: Adjusted for age, sex, education level, occupation, family income, smoking status, alcohol use, BMI, physical activity, household air pollution, passive smoking exposure, temperature, humidity, O_3_, arthritis, diabetes, hypertension, dyslipidaemia, cardiovascular disease, COPD.  *: *P* _FDR_<0.05; **: *P* _FDR_ <0.01; ***: *P* _FDR_ <0.001. | | | | | |

| **Supplementary table 3.** Associations of long-term exposure to 2-year average PM_2.5_ and PM_10_ with relative telomere length (RTL), stratified by demographic variables in South China Cohort (SCC) (N=2023) | | | | | | | |
| --- | --- | --- | --- | --- | --- | --- | --- |
|  | **2-year average PM_2.5_** | | |  | **2-year average PM_10_** | | |
|  | β (95 % CI) | *P* _FDR_ | P-value for interaction |  | β (95 % CI) | *P* _FDR_ | P-value for interaction |
| **All participants** | -0.003 (-0.009, 0.003) | 0.371 | - |  | -0.001 (-0.005, 0.004) | 0.830 | - |
| **Age, years** |  |  | 0.195 |  |  |  | 0.270 |
| <65 | -0.001 (-0.008, 0.006) | 0.733 |  |  | 0.001 (-0.004, 0.005) | 0.836 |  |
| ≥65 | -0.013 (-0.029, 0.003) | 0.119 |  |  | -0.007 (-0.019, 0.005) | 0.262 |  |
| **Sex** |  |  | 0.579 |  |  |  | 0.494 |
| Men | 0.001 (-0.011, 0.012) | 0.997 |  |  | 0.002 (-0.006, 0.01) | 0.629 |  |
| Women | -0.004 (-0.011, 0.004) | 0.306 |  |  | -0.001 (-0.007, 0.004) | 0.600 |  |
| **Education level** |  |  | 0.386 |  |  |  | 0.279 |
| Primary or below | 0.015 (-0.012, 0.043) | 0.279 |  |  | 0.014 (-0.006, 0.034) | 0.178 |  |
| Secondary | -0.004 (-0.011, 0.003) | 0.265 |  |  | -0.002 (-0.007, 0.003) | 0.454 |  |
| College or above | -0.005 (-0.022, 0.013) | 0.620 |  |  | 0.002 (-0.011, 0.015) | 0.755 |  |
| **Marital status** |  |  | 0.356 |  |  |  | 0.391 |
| Never married | 0.048 (-0.015, 0.111) | 0.159 |  |  | 0.035 (-0.011, 0.081) | 0.155 |  |
| Married | -0.004 (-0.01, 0.003) | 0.280 |  |  | -0.001 (-0.006, 0.004) | 0.695 |  |
| Separated/divorced/widowed | 0.001 (-0.028, 0.03) | 0.950 |  |  | 0.001 (-0.02, 0.023) | 0.909 |  |
| **Occupation** |  |  | 0.276 |  |  |  | 0.148 |
| Manual | -0.009 (-0.019, 0.002) | 0.123 |  |  | -0.006 (-0.014, 0.002) | 0.118 |  |
| Non-manual | 0.004 (-0.007, 0.016) | 0.450 |  |  | 0.005 (-0.004, 0.013) | 0.275 |  |
| Other | -0.002 (-0.013, 0.008) | 0.677 |  |  | 0.002 (-0.006, 0.009) | 0.660 |  |
| **Family income, CNY/year** |  |  | 0.937 |  |  |  | 0.937 |
| <50,000 | -0.005 (-0.018, 0.009) | 0.497 |  |  | -0.002 (-0.011, 0.008) | 0.729 |  |
| 50,000–79,999 | 0.001 (-0.012, 0.012) | 0.96 |  |  | 0.002 (-0.006, 0.011) | 0.588 |  |
| ≥80,000 | -0.002 (-0.011, 0.007) | 0.682 |  |  | 0.001 (-0.006, 0.006) | 0.997 |  |
| Don’t know | 0.007 (-0.044, 0.058) | 0.786 |  |  | 0.002 (-0.036, 0.04) | 0.907 |  |
| **Smoking status** |  |  | 0.084 |  |  |  | 0.094 |
| Never | -0.006 (-0.013, 0.001) | 0.102 |  |  | -0.003 (-0.008, 0.002) | 0.305 |  |
| Former | 0.010 (-0.014, 0.033) | 0.422 |  |  | 0.011 (-0.005, 0.027) | 0.169 |  |
| Current | 0.016 (-0.005, 0.036) | 0.133 |  |  | 0.010 (-0.004, 0.025) | 0.171 |  |
| **Alcohol use** |  |  | 0.955 |  |  |  | 0.910 |
| Never | -0.003 (-0.01, 0.004) | 0.406 |  |  | -0.001 (-0.005, 0.004) | 0.837 |  |
| Former | -0.001 (-0.017, 0.017) | 0.992 |  |  | -0.001 (-0.012, 0.013) | 0.993 |  |
| Current | -0.002 (-0.034, 0.031) | 0.917 |  |  | 0.004 (-0.019, 0.027) | 0.731 |  |
| **BMI, kg/m^2^** |  |  | 0.331 |  |  |  | 0.429 |
| <18.5 | 0.004 (-0.034, 0.042) | 0.831 |  |  | 0.009 (-0.019, 0.036) | 0.544 |  |
| 18.5–24.9 | -0.002 (-0.01, 0.005) | 0.528 |  |  | 0.001 (-0.006, 0.005) | 0.927 |  |
| 25.0–27.4 | 0.001 (-0.013, 0.016) | 0.854 |  |  | 0.002 (-0.009, 0.012) | 0.777 |  |
| ≥27.5 | -0.025 (-0.049, -0.001) * | 0.047 |  |  | -0.013 (-0.030, 0.004) | 0.124 |  |
| **Arthritis** |  |  | 0.108 |  |  |  | 0.108 |
| No | -0.001 (-0.008, 0.005) | 0.667 |  |  | 0.001 (-0.004, 0.005) | 0.819 |  |
| Yes | -0.021 (-0.045, 0.002) | 0.076 |  |  | -0.014 (-0.031, 0.003) | 0.112 |  |
| **Diabetes** |  |  | 0.054 |  |  |  | 0.105 |
| No | -0.001 (-0.008, 0.006) | 0.810 |  |  | 0.001 (-0.005, 0.006) | 0.813 |  |
| Yes | -0.016 (-0.029, -0.004) * | 0.013 |  |  | -0.009 (-0.018, 0.001) | 0.066 |  |
| **Hypertension** |  |  | 0.218 |  |  |  | 0.132 |
| No | -0.001 (-0.008, 0.007) | 0.867 |  |  | 0.001 (-0.004, 0.007) | 0.590 |  |
| Yes | -0.009 (-0.021, 0.002) | 0.100 |  |  | -0.006 (-0.015, 0.002) | 0.128 |  |
| **Dyslipidaemia** |  |  | 0.078 |  |  |  | 0.086 |
| No | 0.001 (-0.006, 0.009) | 0.762 |  |  | 0.002 (-0.003, 0.008) | 0.401 |  |
| Yes | -0.011 (-0.022, 0.001) | 0.059 |  |  | -0.006 (-0.014, 0.002) | 0.142 |  |
| **Cardiovascular disease** |  |  | 0.016 |  |  |  | 0.012 |
| No | -0.003 (-0.009, 0.004) | 0.401 |  |  | -0.001 (-0.005, 0.004) | 0.876 |  |
| Yes | -0.310 (-0.514, -0.106) * | 0.013 |  |  | -0.225 (-0.363, -0.086) ** | 0.009 |  |
| PM_2.5_: Particulate matter 2.5; PM_10_: Particulate matter 10; BMI: body mass index; CNY: Chinese yuan.  *: *P* _FDR_<0.05; **: *P* _FDR_ <0.01; ***: *P* _FDR_ <0.001. | | | | | | | |

| **Supplementary table 4.** Associations of long-term exposure to 2-year average PM_2.5_ and PM_10_ with phenotypic age (years) and accelerated age, stratified by PRS in GBCS (N=1604) | | | | |
| --- | --- | --- | --- | --- |
|  | **Phenotypic age (years)** |  | | **Accelerated age** |
|  | β (95 % CI) ^a^ |  | | OR (95 % CI) ^a^ |
| **2-year average PM_2.5_** |  |  |  | |
| Per 1 μg/m^3^ increase in PM_2.5_ | 0.015 (-0.009, 0.040) |  | 1.008 (1.000, 1.016) * | |
| **Stratified by PRS** |  |  |  | |
| **Lower PRS** |  |  |  | |
| Per 1 μg/m^3^ increase in PM_2.5_ | 0.034 (-0.005, 0.073) |  | 1.011 (1.000, 1.023) * | |
| **Higher PRS** |  |  |  | |
| Per 1 μg/m^3^ increase in PM_2.5_ | 0.001 (-0.029, 0.031) |  | 1.005 (0.993, 1.017) | |
| **2-year average PM_10_** |  |  |  | |
| Per 1 μg/m^3^ increase in PM_10_ | 0.010 (-0.008, 0.029) |  | 1.005 (1.000, 1.011) * | |
| **Stratified by PRS** |  |  |  | |
| **Lower PRS** |  |  |  | |
| Per 1 μg/m^3^ increase in PM_10_ | 0.025 (-0.005, 0.055) |  | 1.009 (1.000, 1.018) * | |
| **Higher PRS** |  |  |  | |
| Per 1 μg/m^3^ increase in PM_10_ | 0.001 (-0.024, 0.022) |  | 1.002 (0.994, 1.011) | |
| PM_2.5_: Particulate matter 2.5; PM_10_: Particulate matter 10; PRS: Polygenic risk score; OR: odds ratio; CI: confidence interval.  ^a^: Adjusted for age, sex, education level, occupation, family income, smoking status, alcohol use, BMI, physical activity, household air pollution, passive smoking exposure, temperature, humidity, O_3_, arthritis, diabetes, hypertension, dyslipidaemia, cardiovascular disease, COPD.  *: *P* _FDR_<0.05; **: *P* _FDR_ <0.01; ***: *P* _FDR_ <0.001. | | | | |

| **Supplementary table 5.** Associations of PM _2.5_ and PM _10_ and all-cause mortality in Guangzhou Biobank Cohort Study from 2003 to 2004 and followed up until November 21st, 2023 (N=9527) | | | | | | |
| --- | --- | --- | --- | --- | --- | --- |
| **PM_2.5_** |  | **1-year average** | |  | **2-year average** | |
|  |  | Range (μg/m^3^) | HRs (95% CI) ^a^ |  | Range (μg/m^3^) | HRs (95% CI) ^a^ |
| **Per 1 μg/m^3^ increase in PM_2.5_** |  | 10.54 - 90.99 | 1.087 (1.084, 1.089) *** |  | 11.37 - 92.8 | 1.091 (1.088, 1.093) *** |
| **Stratified by PDI** |  |  |  |  |  |  |
| **Lower PDI** |  |  |  |  |  |  |
| Per 1 μg/m^3^ increase in PM_2.5_ |  | 11.11 - 90.99 | 1.094 (1.091, 1.098) *** |  | 11.37 - 91.4 | 1.098 (1.094, 1.101) *** |
| **Higher PDI** |  |  |  |  |  |  |
| Per 1 μg/m^3^ increase in PM_2.5_ |  | 10.54 - 90.99 | 1.093 (1.089, 1.096) *** |  | 11.48 - 92.8 | 1.097 (1.093, 1.1) *** |
| **Stratified by PHD** |  |  |  |  |  |  |
| **Lower PHD** |  |  |  |  |  |  |
| Per 1 μg/m^3^ increase in PM_2.5_ |  | 10.54 - 90.99 | 1.092 (1.089, 1.096) *** |  | 11.37 - 92.77 | 1.097 (1.094, 1.1) *** |
| **Higher PHD** |  |  |  |  |  |  |
| Per 1 μg/m^3^ increase in PM_2.5_ |  | 11.11 - 90.99 | 1.094 (1.09, 1.097) *** |  | 11.48 - 92.8 | 1.098 (1.094, 1.101) *** |
| **PM_10_** |  | **1-year average** | |  | **2-year average** | |
|  |  | Range (μg/m^3^) | HRs (95% CI) ^a^ |  | Range (μg/m^3^) | HRs (95% CI) ^a^ |
| **Per 1 μg/m^3^ increase in PM_10_** |  | 23.94 - 127.76 | 1.07 (1.068, 1.072) *** |  | 23.67 - 129.28 | 1.074 (1.072, 1.075) *** |
| **Stratified by PDI** |  |  |  |  |  |  |
| **Lower PDI** |  |  |  |  |  |  |
| Per 1 μg/m^3^ increase in PM_10_ |  | 23.94 - 127.76 | 1.075 (1.073, 1.078) *** |  | 23.67 - 129.28 | 1.079 (1.077, 1.082) *** |
| **Higher PDI** |  |  |  |  |  |  |
| Per 1 μg/m^3^ increase in PM_10_ |  | 24.58 - 126.74 | 1.076 (1.074, 1.078) *** |  | 24.29 - 122.87 | 1.079 (1.076, 1.081) *** |
| **Stratified by PHD** |  |  |  |  |  |  |
| **Lower PHD** |  |  |  |  |  |  |
| Per 1 μg/m^3^ increase in PM_10_ |  | 24.15 - 124.31 | 1.074 (1.071, 1.076) *** |  | 23.88 - 125.8 | 1.078 (1.076, 1.08) *** |
| **Higher PHD** |  |  |  |  |  |  |
| Per 1 μg/m^3^ increase in PM_10_ |  | 23.94 - 127.76 | 1.078 (1.075, 1.08) *** |  | 23.67 - 129.28 | 1.080 (1.077, 1.082) *** |
| PM_2.5_: Particulate matter 2.5; PM_10_: Particulate matter 10; PDI: plant-based diets index; PHD: planetary-health diets; HRs: Hazard ratios; CI: confidence interval.  ^a^: Adjusted for age, sex, education level, occupation, family income, smoking status, alcohol use, BMI, physical activity, household air pollution, passive smoking exposure, temperature, humidity, O_3_, arthritis, diabetes, hypertension, dyslipidaemia, cardiovascular disease, COPD.  *: *P* _FDR_<0.05; **: *P* _FDR_ <0.01; ***: *P* _FDR_ <0.001. | | | | | | |

| **Supplementary table 6.** Characteristics of participants in the Guangzhou Biobank Cohort Study | | | | | | | | | | |
| --- | --- | --- | --- | --- | --- | --- | --- | --- | --- | --- |
| Characteristics | Total  (N=9527) | **2-year average PM_2.5_** | | | |  | **2-year average PM_10_** | | | |
|  |  | Tertile 1  (N=3178) | Tertile 2  (N=3174) | Tertile 3  (N=3175) | P value |  | Tertile 1  (N=3177) | Tertile 2  (N=3177) | Tertile 3  (N=3173) | P value |
| **PDI, Median (Q1, Q3)** | 49 (46,52) | 49 (46, 52) | 49 (46, 52) | 49 (46, 53) | 0.004 |  | 49 (46, 52) | 49 (46, 52) | 49 (46, 53) | 0.018 |
| **PHD, Mean ± SD** | 61.2 ± 11.6 | 60.9 ± 11.5 | 60.7 ± 11.6 | 61.9 ± 11.7 | < 0.001 |  | 60.9 ± 11.5 | 60.8 ± 11.5 | 61.8 ± 11.8 | < 0.001 |
| **Temperature,°C, Median (Q1,Q3)** | 22.6 (22.5, 22.7) | 22.6 (22.5, 22.7) | 22.6 (22.5, 22.7) | 22.6 (22.5, 22.7) | < 0.001 |  | 22.6 (22.5, 22.7) | 22.6 (22.5, 22.7) | 22.6 (22.5, 22.7) | < 0.001 |
| **Humidity, %, Median (Q1,Q3)** | 72 (71.7, 72) | 72 (71.9, 72.2) | 72 (71.8, 72) | 72 (71.4, 72) | < 0.001 |  | 72 (71.9, 72.2) | 72 (71.9, 72) | 72 (71.4, 72) | < 0.001 |
| **O_3_, μg/m³, Median (Q1,Q3)** | 94.7 (80.7, 112) | 106.9 (96, 116.2) | 95.3 (84.7, 110.9) | 78.2 (71.7, 90) | < 0.001 |  | 106.8 (95.9, 116.6) | 94.8 (83.2, 110.3) | 78.6 (71.7, 91.8) | < 0.001 |
| **Age, years, %** |  |  |  |  | < 0.001 |  |  |  |  | < 0.001 |
| <65 | 4970 (52.2) | 1707 (53.7) | 1744 (54.9) | 1519 (47.8) |  |  | 1709 (53.8) | 1741 (54.8) | 1520 (47.9) |  |
| ≥65 | 4557 (47.8) | 1471 (46.3) | 1430 (45.1) | 1656 (52.2) |  |  | 1468 (46.2) | 1436 (45.2) | 1653 (52.1) |  |
| **Sex, %** |  |  |  |  | < 0.001 |  |  |  |  | < 0.001 |
| Men | 2787 (29.3) | 1028 (32.3) | 951 (30.0) | 808 (25.4) |  |  | 1015 (31.9) | 970 (30.5) | 802 (25.3) |  |
| Women | 6740 (70.7) | 2150 (67.7) | 2223 (70.0) | 2367 (74.6) |  |  | 2162 (68.1) | 2207 (69.5) | 2371 (74.7) |  |
| **Education level, %** |  |  |  |  | 0.003 |  |  |  |  | 0.028 |
| Primary or below | 4776 (50.1) | 1509 (47.5) | 1629 (51.3) | 1638 (51.6) |  |  | 1527 (48.1) | 1605 (50.5) | 1644 (51.8) |  |
| Secondary | 3887 (40.8) | 1346 (42.4) | 1276 (40.2) | 1265 (39.8) |  |  | 1334 (42.0) | 1292 (40.7) | 1261 (39.7) |  |
| College or above | 864 (9.1) | 323 (10.1) | 269 (8.5) | 272 (8.6) |  |  | 316 (9.9) | 280 (8.8) | 268 (8.5) |  |
| **Occupation, %** |  |  |  |  | 0.011 |  |  |  |  | 0.020 |
| Manual | 2963 (31.1) | 1058 (33.3) | 924 (29.1) | 981 (30.9) |  |  | 1045 (32.9) | 949 (29.9) | 969 (30.5) |  |
| Non-manual | 6095 (64.0) | 1970 (62.0) | 2087 (65.8) | 2038 (64.2) |  |  | 1987 (62.5) | 2049 (64.5) | 2059 (64.9) |  |
| Other | 469 (4.9) | 150 (4.7) | 163 (5.1) | 156 (4.9) |  |  | 145 (4.6) | 179 (5.6) | 145 (4.6) |  |
| **Family income, CNY/year, %** |  |  |  |  | 0.004 |  |  |  |  | 0.016 |
| <10,000 | 663 (7.0) | 212 (6.7) | 230 (7.2) | 221 (7.0) |  |  | 219 (6.9) | 221 (7.0) | 223 (7.0) |  |
| 10,000–29,999 | 3205 (33.6) | 1113 (35.0) | 1034 (32.6) | 1058 (33.3) |  |  | 1113 (35.0) | 1038 (32.7) | 1054 (33.2) |  |
| 30,000–49,999 | 1452 (15.2) | 525 (16.5) | 482 (15.2) | 445 (14.0) |  |  | 514 (16.2) | 489 (15.4) | 449 (14.2) |  |
| ≥50,000 | 992 (10.4) | 343 (10.8) | 332 (10.5) | 317 (10.0) |  |  | 338 (10.6) | 342 (10.8) | 312 (9.8) |  |
| Don’t know | 3215 (33.8) | 985 (31.0) | 1096 (34.5) | 1134 (35.7) |  |  | 993 (31.3) | 1087 (34.1) | 1135 (35.8) |  |
| **Smoking status, %** |  |  |  |  | 0.014 |  |  |  |  | 0.006 |
| Never | 7565 (79.4) | 2478 (78.0) | 2503 (78.9) | 2584 (81.4) |  |  | 2479 (78.0) | 2498 (78.6) | 2588 (81.6) |  |
| Former | 1010 (10.6) | 354 (11.1) | 348 (11.0) | 308 (9.7) |  |  | 353 (11.1) | 350 (11.0) | 307 (9.7) |  |
| Current | 952 (10.0) | 346 (10.9) | 323 (10.1) | 283 (8.9) |  |  | 345 (10.9) | 329 (10.4) | 278 (8.7) |  |
| **Alcohol use, %** |  |  |  |  | 0.475 |  |  |  |  | 0.297 |
| Never | 7950 (83.4) | 2674 (84.1) | 2633 (83.0) | 2643 (83.2) |  |  | 2676 (84.2) | 2621 (82.5) | 2653 (83.6) |  |
| Former | 206 (2.2) | 61 (2.0) | 79 (2.5) | 66 (2.1) |  |  | 62 (2.0) | 80 (2.5) | 64 (2.0) |  |
| Current | 1371 (14.4) | 443 (13.9) | 462 (14.5) | 466 (14.7) |  |  | 439 (13.8) | 476 (15.0) | 456 (14.4) |  |
| **BMI, kg/m^2^, %** |  |  |  |  | 0.409 |  |  |  |  | 0.116 |
| <18.5 | 460 (4.8) | 163 (5.1) | 154 (4.9) | 143 (4.5) |  |  | 159 (5.0) | 160 (5.0) | 141 (4.4) |  |
| 18.5–24.9 | 5798 (60.9) | 1970 (62.0) | 1919 (60.5) | 1909 (60.1) |  |  | 1993 (62.7) | 1895 (59.6) | 1910 (60.2) |  |
| 25.0–27.4 | 2057 (21.6) | 666 (21.0) | 695 (21.9) | 696 (21.9) |  |  | 647 (20.4) | 708 (22.3) | 702 (22.1) |  |
| ≥27.5 | 1212 (12.7) | 379 (11.9) | 406 (12.7) | 427 (13.5) |  |  | 378 (11.9) | 414 (13.1) | 420 (13.3) |  |
| **Physical activity, %** |  |  |  |  | < 0.001 |  |  |  |  | < 0.001 |
| Inactive | 137 (1.4) | 48 (1.5) | 42 (1.3) | 47 (1.5) |  |  | 45 (1.4) | 42 (1.3) | 50 (1.6) |  |
| Moderate | 3325 (34.9) | 1038 (32.7) | 1058 (33.3) | 1229 (38.7) |  |  | 1039 (32.7) | 1053 (33.1) | 1233 (38.9) |  |
| Active | 6065 (63.7) | 2092 (65.8) | 2074 (65.4) | 1899 (59.8) |  |  | 2093 (65.9) | 2082 (65.6) | 1890 (59.5) |  |
| **Household air pollution, %** |  |  |  |  | 0.896 |  |  |  |  | 0.966 |
| No | 9405 (98.7) | 3139 (98.8) | 3131 (98.6) | 3135 (98.7) |  |  | 3137 (98.7) | 3137 (98.7) | 3131 (98.7) |  |
| Yes | 122 (1.3) | 39 (1.2) | 43 (1.4) | 40 (1.3) |  |  | 40 (1.3) | 40 (1.3) | 42 (1.3) |  |
| **Passive smoking exposure, %** |  |  |  |  | < 0.001 |  |  |  |  | < 0.001 |
| <2 years of 40 h per week | 1582 (16.6) | 571 (18.0) | 550 (17.3) | 461 (14.5) |  |  | 576 (18.1) | 542 (17.1) | 464 (14.6) |  |
| 2–5 years of 40 h per week | 1157 (12.1) | 325 (10.2) | 360 (11.3) | 472 (14.9) |  |  | 332 (10.5) | 349 (11.0) | 476 (15.0) |  |
| >5 years of 40 h per week | 1953 (20.5) | 588 (18.5) | 649 (20.4) | 716 (22.6) |  |  | 591 (18.6) | 642 (20.2) | 720 (22.7) |  |
| Not reported | 4835 (50.8) | 1694 (53.3) | 1615 (51.0) | 1526 (48.0) |  |  | 1678 (52.8) | 1644 (51.7) | 1513 (47.7) |  |
| **Arthritis, %** |  |  |  |  | < 0.001 |  |  |  |  | < 0.001 |
| No | 7164 (75.2) | 2255 (71.0) | 2389 (75.3) | 2520 (79.4) |  |  | 2258 (71.1) | 2373 (74.7) | 2533 (79.8) |  |
| Yes | 2363 (24.8) | 923 (29.0) | 785 (24.7) | 655 (20.6) |  |  | 919 (28.9) | 804 (25.3) | 640 (20.2) |  |
| **Diabetes, %** |  |  |  |  | 0.030 |  |  |  |  | 0.070 |
| No | 8656 (90.9) | 2881 (90.7) | 2917 (91.9) | 2858 (90.0) |  |  | 2879 (90.6) | 2916 (91.8) | 2861 (90.2) |  |
| Yes | 871 (9.1) | 297 (9.3) | 257 (8.1) | 317 (10.0) |  |  | 298 (9.4) | 261 (8.2) | 312 (9.8) |  |
| **Hypertension, %** |  |  |  |  | 0.123 |  |  |  |  | 0.281 |
| No | 6485 (68.1) | 2147 (67.6) | 2204 (69.4) | 2134 (67.2) |  |  | 2152 (67.7) | 2196 (69.1) | 2137 (67.3) |  |
| Yes | 3042 (31.9) | 1031 (32.4) | 970 (30.6) | 1041 (32.8) |  |  | 1025 (32.3) | 981 (30.9) | 1036 (32.7) |  |
| **Dyslipidaemia, %** |  |  |  |  | 0.300 |  |  |  |  | 0.326 |
| No | 8656 (90.9) | 2870 (90.3) | 2902 (91.4) | 2884 (90.8) |  |  | 2868 (90.3) | 2902 (91.3) | 2886 (91.0) |  |
| Yes | 871 (9.1) | 308 (9.7) | 272 (8.6) | 291 (9.2) |  |  | 309 (9.7) | 275 (8.7) | 287 (9.0) |  |
| **Cardiovascular disease, %** |  |  |  |  | 0.443 |  |  |  |  | 0.560 |
| No | 8925 (93.7) | 2965 (93.3) | 2986 (94.1) | 2974 (93.7) |  |  | 2968 (93.4) | 2988 (94.1) | 2969 (93.6) |  |
| Yes | 602 (6.3) | 213 (6.7) | 188 (5.9) | 201 (6.3) |  |  | 209 (6.6) | 189 (5.9) | 204 (6.4) |  |
| **COPD, %** |  |  |  |  | 0.008 |  |  |  |  | 0.384 |
| No | 7768 (81.5) | 2585 (81.3) | 2639 (83.1) | 2544 (80.1) |  |  | 2585 (81.4) | 2614 (82.3) | 2569 (81) |  |
| Yes | 1759 (18.5) | 593 (18.7) | 535 (16.9) | 631 (19.9) |  |  | 592 (18.6) | 563 (17.7) | 604 (19) |  |
| PDI: plant-based diets index; PHD: planetary-health diets; Q1: Quartile 1; Q3: Quartile 3; SD: Standard deviation; PM_2.5_: Particulate matter 2.5; PM_10_: Particulate matter 10; O_3_: ozone; BMI: body mass index; CNY: Chinese yuan; COPD: chronic obstructive pulmonary disease. | | | | | | | | | | |

| **Supplementary table 7.** Associations of long-term exposure to 1-year average PM_2.5_ with phenotypic age (years) and accelerated age**,** stratified by demographic variables in GBCS (N=9527) | | | | | | | |  |
| --- | --- | --- | --- | --- | --- | --- | --- | --- |
|  | **Phenotypic age (years)** | | |  | **Accelerated age** | | | |
|  | β (95 % CI) | *P* _FDR_ | P-value for interaction |  | OR (95 % CI) | *P* _FDR_ | P-value for interaction | |
| **Age, years** |  |  | 0.431 |  |  |  | 0.520 | |
| <65 | 0.027 (0.012, 0.042) *** | <0.001 |  |  | 1.004 (1.000, 1.008) | 0.062 |  | |
| ≥65 | 0.035 (0.020, 0.051) *** | <0.001 |  |  | 1.006 (1.002, 1.010) ** | 0.006 |  | |
| **Sex** |  |  | 0.646 |  |  |  | 0.398 | |
| Men | 0.056 (0.032, 0.079) *** | <0.001 |  |  | 1.01 (1.004, 1.016) ** | 0.001 |  | |
| Women | 0.062 (0.047, 0.078) *** | <0.001 |  |  | 1.007 (1.003, 1.011) *** | <0.001 |  | |
| **Education level** |  |  | 0.770 |  |  |  | 0.670 | |
| Primary or below | 0.050 (0.031, 0.069) *** | <0.001 |  |  | 1.006 (1.002, 1.011) ** | 0.003 |  | |
| Secondary | 0.040 (0.019, 0.060) *** | <0.001 |  |  | 1.004 (0.999, 1.008) | 0.115 |  | |
| College or above | 0.049 (0.006, 0.092) * | 0.027 |  |  | 1.004 (0.994, 1.014) | 0.431 |  | |
| **Occupation** |  |  | 0.614 |  |  |  | 0.709 | |
| Manual | 0.046 (0.022, 0.07) *** | <0.001 |  |  | 1.007 (1.002, 1.012) * | 0.011 |  | |
| Non-manual | 0.051 (0.035, 0.068) *** | <0.001 |  |  | 1.004 (1.000, 1.008) * | 0.030 |  | |
| Other | 0.019 (-0.046, 0.084) | 0.575 |  |  | 1.005 (0.991, 1.019) | 0.503 |  | |
| **Family income, CNY/year** |  |  | 0.808 |  |  |  | 0.838 | |
| <10,000 | 0.068 (0.018, 0.117) ** | 0.007 |  |  | 1.002 (0.991, 1.013) | 0.737 |  | |
| 10,000–29,999 | 0.050 (0.026, 0.073) *** | <0.001 |  |  | 1.004 (0.999, 1.009) | 0.143 |  | |
| 30,000–49,999 | 0.040 (0.006, 0.074) * | 0.020 |  |  | 1.005 (0.998, 1.013) | 0.155 |  | |
| ≥50,000 | 0.056 (0.014, 0.097) ** | 0.009 |  |  | 1.010 (1.000, 1.019) * | 0.043 |  | |
| Don’t know | 0.038 (0.015, 0.060) ** | 0.001 |  |  | 1.005 (1.000, 1.010) | 0.051 |  | |
| **Smoking status** |  |  | 0.001 |  |  |  | 0.163 | |
| Never | 0.053 (0.038, 0.068) *** | <0.001 |  |  | 1.006 (1.003, 1.010) *** | <0.001 |  | |
| Former | 0.110 (0.072, 0.149) *** | <0.001 |  |  | 1.012 (1.002, 1.021) * | 0.014 |  | |
| Current | -0.004 (-0.043, 0.036) | 0.861 |  |  | 1.000 (0.989, 1.008) | 0.773 |  | |
| **Alcohol use** |  |  | 0.330 |  |  |  | 0.691 | |
| Never | 0.051 (0.037, 0.066) *** | <0.001 |  |  | 1.004 (1.001, 1.008) * | 0.011 |  | |
| Former | 0.015 (-0.077, 0.106) | 0.756 |  |  | 1.004 (0.984, 1.025) | 0.695 |  | |
| Current | 0.026 (-0.008, 0.061) | 0.136 |  |  | 1.008 (1.000, 1.016) * | 0.043 |  | |
| **BMI, kg/m^2^** |  |  | 0.326 |  |  |  | 0.290 | |
| <18.5 | 0.009 (-0.053, 0.071) | 0.776 |  |  | 0.990 (0.976, 1.005) | 0.186 |  | |
| 18.5–24.9 | 0.050 (0.033, 0.067) *** | <0.001 |  |  | 1.005 (1.001, 1.009) * | 0.015 |  | |
| 25.0–27.4 | 0.030 (0.001, 0.060) * | 0.045 |  |  | 1.005 (0.998, 1.011) | 0.150 |  | |
| ≥27.5 | 0.061 (0.023, 0.099) ** | 0.002 |  |  | 1.005 (0.997, 1.013) | 0.240 |  | |
| **Physical activity** |  |  | 0.219 |  |  |  | 0.685 | |
| Inactive | 0.128 (0.008, 0.247) * | 0.038 |  |  | 1.009 (0.987, 1.032) | 0.411 |  | |
| Moderate | 0.046 (0.023, 0.069) *** | <0.001 |  |  | 1.006 (1.001, 1.011) * | 0.020 |  | |
| Active | 0.038 (0.022, 0.055) *** | <0.001 |  |  | 1.003 (1.000, 1.007) | 0.079 |  | |
| **Arthritis** |  |  | 0.569 |  |  |  | 0.311 | |
| No | 0.039 (0.011, 0.067) ** | 0.007 |  |  | 1.004 (1.000, 1.007) * | 0.030 |  | |
| Yes | 0.048 (0.033, 0.063) *** | <0.001 |  |  | 1.007 (1.001, 1.014) * | 0.019 |  | |
| **Household air pollution** |  |  | 0.134 |  |  |  | 0.333 | |
| No | 0.046 (0.033, 0.060) *** | <0.001 |  |  | 1.005 (1.002, 1.008) ** | 0.002 |  | |
| Yes | 0.144 (0.009, 0.279) * | 0.039 |  |  | 1.019 (0.991, 1.048) | 0.193 |  | |
| **Passive smoking exposure** |  |  | 0.530 |  |  |  | 0.540 | |
| <2 years of 40 h per week | 0.065 (0.033, 0.098) *** | <0.001 |  |  | 1.010 (1.002, 1.017) * | 0.011 |  | |
| 2–5 years of 40 h per week | 0.058 (0.020, 0.095) ** | 0.003 |  |  | 1.003 (0.994, 1.011) | 0.513 |  | |
| >5 years of 40 h per week | 0.056 (0.027, 0.085) *** | <0.001 |  |  | 1.007 (1.000, 1.013) * | 0.042 |  | |
| Not reported | 0.040 (0.021, 0.059) *** | <0.001 |  |  | 1.004 (1.000, 1.008) | 0.053 |  | |
| **Diabetes** |  |  | 0.428 |  |  |  | 0.927 | |
| No | 0.045 (0.031, 0.058) *** | <0.001 |  |  | 1.005 (1.002, 1.008) ** | 0.003 |  | |
| Yes | 0.063 (0.013, 0.112) * | 0.014 |  |  | 1.005 (0.994, 1.017) | 0.348 |  | |
| **Hypertension** |  |  | 0.401 |  |  |  | 0.435 | |
| No | 0.043 (0.027, 0.058) *** | <0.001 |  |  | 1.004 (1.000, 1.008) * | 0.031 |  | |
| Yes | 0.055 (0.030, 0.080) *** | <0.001 |  |  | 1.007 (1.001, 1.012) * | 0.014 |  | |
| **Dyslipidaemia** |  |  | 0.236 |  |  |  | 0.951 | |
| No | 0.045 (0.031, 0.059) *** | <0.001 |  |  | 1.005 (1.002, 1.008) ** | 0.002 |  | |
| Yes | 0.073 (0.027, 0.118) ** | 0.002 |  |  | 1.005 (0.996, 1.015) | 0.296 |  | |
| **Cardiovascular disease** |  |  | 0.740 |  |  |  | 0.241 | |
| No | 0.048 (0.034, 0.062) *** | <0.001 |  |  | 1.005 (1.002, 1.008) ** | 0.001 |  | |
| Yes | 0.039 (-0.017, 0.094) | 0.174 |  |  | 0.998 (0.987, 1.010) | 0.756 |  | |
| **COPD, %** |  |  | 0.360 |  |  |  | 0.011 | |
| No | 0.05 (0.035, 0.065) *** | <0.001 |  |  | 1.005 (1.002, 1.007) *** | <0.001 |  | |
| Yes | 0.034 (0.002, 0.066) * | 0.038 |  |  | 0.997 (0.992, 1.002) | 0.289 |  | |
| PM_2.5_: Particulate matter 2.5; BMI: body mass index; CNY: Chinese yuan; OR: odds ratio; CI: confidence interval; COPD: chronic obstructive pulmonary disease.  *: *P* _FDR_<0.05; **: *P* _FDR_ <0.01; ***: *P* _FDR_ <0.001. | | | | | | | |  |

| **Supplementary table 8.** Associations of long-term exposure to 1-year average PM_10_ with phenotypic age (years) and accelerated age**,** stratified by demographic variables in GBCS (N=9527) | | | | | | | |
| --- | --- | --- | --- | --- | --- | --- | --- |
|  | Phenotypic age (years) | | |  | Accelerated age | | |
|  | β (95 % CI) | *P* _FDR_ | P-value for interaction |  | OR (95 % CI) | *P* _FDR_ | P-value for interaction |
| **Age, years** |  |  | 0.361 |  |  |  | 0.578 |
| <65 | 0.020 (0.008, 0.031) ** | 0.001 |  |  | 1.003 (1.000, 1.006) | 0.084 |  |
| ≥65 | 0.027 (0.015, 0.039) *** | <0.001 |  |  | 1.004 (1.001, 1.007) * | 0.013 |  |
| **Sex** |  |  | 0.740 |  |  |  | 0.418 |
| Men | 0.044 (0.026, 0.061) *** | <0.001 |  |  | 1.007 (1.003, 1.011) ** | 0.001 |  |
| Women | 0.047 (0.035, 0.059) *** | <0.001 |  |  | 1.005 (1.002, 1.008) *** | <0.001 |  |
| **Education level** |  |  | 0.747 |  |  |  | 0.606 |
| Primary or below | 0.039 (0.024, 0.053) *** | <0.001 |  |  | 1.005 (1.002, 1.008) ** | 0.004 |  |
| Secondary | 0.031 (0.015, 0.047) *** | <0.001 |  |  | 1.002 (0.999, 1.006) | 0.188 |  |
| College or above | 0.036 (0.003, 0.069) * | 0.032 |  |  | 1.003 (0.995, 1.010) | 0.491 |  |
| **Occupation** |  |  | 0.582 |  |  |  | 0.718 |
| Manual | 0.036 (0.018, 0.054) *** | <0.001 |  |  | 1.005 (1.001, 1.009) * | 0.018 |  |
| Non-manual | 0.040 (0.027, 0.052) *** | <0.001 |  |  | 1.003 (1.000, 1.006) | 0.051 |  |
| Other | 0.012 (-0.039, 0.063) | 0.642 |  |  | 1.003 (0.992, 1.015) | 0.565 |  |
| **Family income, CNY/year** |  |  | 0.775 |  |  |  | 0.779 |
| <10,000 | 0.054 (0.016, 0.092) ** | 0.006 |  |  | 1.002 (0.993, 1.010) | 0.709 |  |
| 10,000–29,999 | 0.038 (0.02, 0.056) *** | <0.001 |  |  | 1.002 (0.998, 1.006) | 0.289 |  |
| 30,000–49,999 | 0.029 (0.003, 0.055) * | 0.028 |  |  | 1.004 (0.998, 1.010) | 0.168 |  |
| ≥50,000 | 0.044 (0.013, 0.076) ** | 0.006 |  |  | 1.007 (1.000, 1.014) * | 0.047 |  |
| Don’t know | 0.030 (0.013, 0.048) ** | 0.001 |  |  | 1.004 (1.000, 1.008) | 0.056 |  |
| **Smoking status** |  |  | 0.001 |  |  |  | 0.209 |
| Never | 0.041 (0.030, 0.052) *** | <0.001 |  |  | 1.005 (1.002, 1.007) ** | 0.001 |  |
| Former | 0.082 (0.053, 0.111) *** | <0.001 |  |  | 1.008 (1.001, 1.015) * | 0.031 |  |
| Current | -0.002 (-0.032, 0.028) | 0.880 |  |  | 0.999 (0.991, 1.006) | 0.726 |  |
| **Alcohol use** |  |  | 0.401 |  |  |  | 0.686 |
| Never | 0.039 (0.028, 0.05) *** | <0.001 |  |  | 1.003 (1.000, 1.005) * | 0.022 |  |
| Former | 0.014 (-0.057, 0.084) | 0.707 |  |  | 1.002 (0.987, 1.018) | 0.776 |  |
| Current | 0.022 (-0.004, 0.048) | 0.103 |  |  | 1.006 (1.000, 1.012) | 0.056 |  |
| **BMI, kg/m^2^** |  |  | 0.451 |  |  |  | 0.353 |
| <18.5 | 0.007 (-0.041, 0.055) | 0.769 |  |  | 0.993 (0.982, 1.004) | 0.208 |  |
| 18.5–24.9 | 0.038 (0.025, 0.051) *** | <0.001 |  |  | 1.003 (1.000, 1.006) * | 0.035 |  |
| 25.0–27.4 | 0.027 (0.005, 0.050) * | 0.019 |  |  | 1.004 (0.999, 1.009) | 0.136 |  |
| ≥27.5 | 0.046 (0.018, 0.075) ** | 0.002 |  |  | 1.003 (0.997, 1.010) | 0.330 |  |
| **Physical activity** |  |  | 0.226 |  |  |  | 0.636 |
| Inactive | 0.097 (0.004, 0.190) * | 0.043 |  |  | 1.007 (0.990, 1.025) | 0.421 |  |
| Moderate | 0.036 (0.019, 0.054) *** | <0.001 |  |  | 1.004 (1.000, 1.008) * | 0.028 |  |
| Active | 0.029 (0.017, 0.042) *** | <0.001 |  |  | 1.002 (0.999, 1.005) | 0.139 |  |
| **Arthritis** |  |  | 0.606 |  |  |  | 0.302 |
| No | 0.037 (0.025, 0.049) *** | <0.001 |  |  | 1.005 (1.001, 1.010) * | 0.027 |  |
| Yes | 0.031 (0.009, 0.052) ** | 0.005 |  |  | 1.003 (1.000, 1.005) | 0.055 |  |
| **Household air pollution** |  |  | 0.116 |  |  |  | 0.302 |
| No | 0.036 (0.025, 0.046) *** | <0.001 |  |  | 1.003 (1.000, 1.005) | 0.055 |  |
| Yes | 0.111 (0.012, 0.210) * | 0.030 |  |  | 1.005 (1.001, 1.010) * | 0.027 |  |
| **Passive smoking exposure** |  |  | 0.562 |  |  |  | 0.609 |
| <2 years of 40 h per week | 0.048 (0.023, 0.073) *** | <0.001 |  |  | 1.007 (1.001, 1.012) * | 0.024 |  |
| 2–5 years of 40 h per week | 0.044 (0.015, 0.073) ** | 0.003 |  |  | 1.002 (0.996, 1.009) | 0.536 |  |
| >5 years of 40 h per week | 0.045 (0.022, 0.067) *** | <0.001 |  |  | 1.005 (1.000, 1.010) | 0.051 |  |
| Not reported | 0.031 (0.017, 0.046) *** | <0.001 |  |  | 1.003 (1.000, 1.006) | 0.085 |  |
| **Diabetes** |  |  | 0.349 |  |  |  | 0.867 |
| No | 0.034 (0.024, 0.045) *** | <0.001 |  |  | 1.003 (1.001, 1.006) ** | 0.007 |  |
| Yes | 0.051 (0.013, 0.089) ** | 0.009 |  |  | 1.004 (0.996, 1.013) | 0.350 |  |
| **Hypertension** |  |  | 0.406 |  |  |  | 0.429 |
| No | 0.033 (0.021, 0.045) *** | <0.001 |  |  | 1.003 (1.000, 1.005) | 0.056 |  |
| Yes | 0.042 (0.023, 0.061) *** | <0.001 |  |  | 1.005 (1.001, 1.009) * | 0.022 |  |
| **Dyslipidaemia** |  |  | 0.230 |  |  |  | 0.770 |
| No | 0.035 (0.024, 0.045) *** | <0.001 |  |  | 1.003 (1.001, 1.006) ** | 0.006 |  |
| Yes | 0.057 (0.022, 0.092) ** | 0.002 |  |  | 1.004 (0.997, 1.012) | 0.240 |  |
| **Cardiovascular disease** |  |  | 0.755 |  |  |  | 0.174 |
| No | 0.037 (0.026, 0.047) *** | <0.001 |  |  | 1.004 (1.001, 1.006) ** | 0.001 |  |
| Yes | 0.030 (-0.012, 0.073) | 0.163 |  |  | 0.997 (0.989, 1.006) | 0.574 |  |
| **COPD, %** |  |  | 0.620 |  |  |  | 0.011 |
| No | 0.038 (0.027, 0.049) *** | <0.001 |  |  | 1.005 (1.002, 1.007) *** | <0.001 |  |
| Yes | 0.031 (0.006, 0.056) * | 0.014 |  |  | 0.997 (0.992, 1.002) | 0.289 |  |
| PM_10_: Particulate matter 10; BMI: body mass index; CNY: Chinese yuan; OR: odds ratio; CI: confidence interval; COPD: chronic obstructive pulmonary disease.  *: *P* _FDR_<0.05; **: *P* _FDR_ <0.01; ***: *P* _FDR_ <0.001. | | | | | | | |

| **Supplementary table 9.** Associations of long-term exposure to 2-year average PM_2.5_ with phenotypic age (years) and accelerated age**,** stratified by demographic variables in GBCS (N=9527) | | | | | | | |
| --- | --- | --- | --- | --- | --- | --- | --- |
|  | Phenotypic age (years) | | |  | Accelerated age | | |
|  | β (95 % CI) | *P* _FDR_ | P-value for interaction |  | OR (95 % CI) | *P* _FDR_ | P-value for interaction |
| **Age, years** |  |  | 0.533 |  |  |  | 0.489 |
| <65 | 0.024 (0.009, 0.039) ** | 0.002 |  |  | 1.004 (0.999, 1.008) | 0.101 |  |
| ≥65 | 0.031 (0.015, 0.047) *** | <0.001 |  |  | 1.006 (1.001, 1.010) * | 0.010 |  |
| **Sex** |  |  | 0.799 |  |  |  | 0.394 |
| Men | 0.060 (0.036, 0.084) *** | <0.001 |  |  | 1.010 (1.004, 1.016) ** | 0.001 |  |
| Women | 0.056 (0.041, 0.072)  *** | <0.001 |  |  | 1.007 (1.003, 1.011) *** | <0.001 |  |
| **Education level** |  |  | 0.974 |  |  |  | 0.676 |
| Primary or below | 0.043 (0.024, 0.063) *** | <0.001 |  |  | 1.006 (1.002, 1.010) ** | 0.005 |  |
| Secondary | 0.041 (0.019, 0.062) *** | <0.001 |  |  | 1.004 (0.999, 1.008) | 0.140 |  |
| College or above | 0.045 (0.001, 0.089) * | 0.046 |  |  | 1.003 (0.993, 1.013) | 0.555 |  |
| **Occupation** |  |  | 0.599 |  |  |  | 0.86 |
| Manual | 0.043 (0.018, 0.068) ** | 0.001 |  |  | 1.006 (1.000, 1.011) * | 0.037 |  |
| Non-manual | 0.048 (0.031, 0.065) *** | <0.001 |  |  | 1.004 (1.001, 1.008) * | 0.024 |  |
| Other | 0.014 (-0.052, 0.08) | 0.677 |  |  | 1.002 (0.987, 1.017) | 0.802 |  |
| **Family income, CNY/year** |  |  | 0.897 |  |  |  | 0.878 |
| <10,000 | 0.066 (0.015, 0.116) * | 0.011 |  |  | 1.001 (0.989, 1.012) | 0.911 |  |
| 10,000–29,999 | 0.044 (0.02, 0.068) *** | <0.001 |  |  | 1.004 (0.999, 1.009) | 0.144 |  |
| 30,000–49,999 | 0.034 (0.001, 0.069) | 0.053 |  |  | 1.004 (0.996, 1.012) | 0.329 |  |
| ≥50,000 | 0.047 (0.005, 0.089) * | 0.030 |  |  | 1.008 (0.999, 1.018) | 0.096 |  |
| Don’t know | 0.040 (0.017, 0.063) ** | 0.001 |  |  | 1.006 (1.000, 1.011) * | 0.036 |  |
| **Smoking status** |  |  | 0.001 |  |  |  | 0.390 |
| Never | 0.048 (0.033, 0.063) *** | <0.001 |  |  | 1.006 (1.002, 1.009) ** | 0.001 |  |
| Former | 0.113 (0.073, 0.153) *** | <0.001 |  |  | 1.011 (1.001, 1.020) * | 0.025 |  |
| Current | 0.004 (-0.036, 0.045) | 0.841 |  |  | 1.001 (0.991, 1.011) | 0.817 |  |
| **Alcohol use** |  |  | 0.539 |  |  |  | 0.828 |
| Never | 0.048 (0.033, 0.062) *** | <0.001 |  |  | 1.004 (1.001, 1.008) * | 0.014 |  |
| Former | 0.021 (-0.072, 0.113) | 0.658 |  |  | 1.008 (0.987, 1.029) | 0.470 |  |
| Current | 0.028 (-0.008, 0.064) | 0.123 |  |  | 1.007 (0.999, 1.014) | 0.103 |  |
| **BMI, kg/m^2^** |  |  | 0.294 |  |  |  | 0.315 |
| <18.5 | 0.013 (-0.050, 0.076) | 0.694 |  |  | 0.990 (0.976, 1.005) | 0.207 |  |
| 18.5–24.9 | 0.046 (0.029, 0.064) *** | <0.001 |  |  | 1.004 (1.000, 1.008) * | 0.035 |  |
| 25.0–27.4 | 0.025 (-0.006, 0.055) | 0.113 |  |  | 1.006 (0.999, 1.012) | 0.090 |  |
| ≥27.5 | 0.064 (0.026, 0.103) ** | 0.001 |  |  | 1.005 (0.996, 1.013) | 0.259 |  |
| **Physical activity** |  |  | 0.095 |  |  |  | 0.674 |
| Inactive | 0.140 (0.017, 0.262) * | 0.027 |  |  | 1.011 (0.987, 1.034) | 0.372 |  |
| Moderate | 0.048 (0.025, 0.072) *** | <0.001 |  |  | 1.005 (1.001, 1.010) * | 0.030 |  |
| Active | 0.033 (0.016, 0.05) *** | <0.001 |  |  | 1.003 (0.999, 1.007) | 0.104 |  |
| **Arthritis** |  |  | 0.400 |  |  |  | 0.382 |
| No | 0.046 (0.030, 0.061) *** | <0.001 |  |  | 1.004 (1.000, 1.007) * | 0.045 |  |
| Yes | 0.032 (0.003, 0.061) * | 0.031 |  |  | 1.007 (1.000, 1.013) * | 0.037 |  |
| **Household air pollution** |  |  | 0.368 |  |  |  | 0.621 |
| No | 0.043 (0.030, 0.057) *** | <0.001 |  |  | 1.004 (1.001, 1.008) ** | 0.004 |  |
| Yes | 0.103 (-0.036, 0.242) | 0.148 |  |  | 1.012 (0.983, 1.041) | 0.421 |  |
| **Passive smoking exposure** |  |  | 0.498 |  |  |  | 0.601 |
| <2 years of 40 h per week | 0.064 (0.030, 0.097) *** | <0.001 |  |  | 1.008 (1.000, 1.016) * | 0.039 |  |
| 2–5 years of 40 h per week | 0.050 (0.012, 0.089) * | 0.010 |  |  | 1.004 (0.995, 1.013) | 0.369 |  |
| >5 years of 40 h per week | 0.056 (0.026, 0.086) *** | <0.001 |  |  | 1.008 (1.001, 1.015) * | 0.022 |  |
| Not reported | 0.037 (0.017, 0.056) *** | <0.001 |  |  | 1.003 (0.999, 1.008) | 0.111 |  |
| **Diabetes** |  |  | 0.665 |  |  |  | 0.985 |
| No | 0.041 (0.027, 0.055) *** | <0.001 |  |  | 1.004 (1.001, 1.008) ** | 0.007 |  |
| Yes | 0.051 (0.001, 0.102) * | 0.047 |  |  | 1.004 (0.993, 1.016) | 0.458 |  |
| **Hypertension** |  |  | 0.300 |  |  |  | 0.218 |
| No | 0.038 (0.022, 0.054) *** | <0.001 |  |  | 1.003 (0.999, 1.007) | 0.093 |  |
| Yes | 0.054 (0.028, 0.079) *** | <0.001 |  |  | 1.007 (1.002, 1.013) ** | 0.008 |  |
| **Dyslipidaemia** |  |  | 0.119 |  |  |  | 0.768 |
| No | 0.04 (0.026, 0.055) *** | <0.001 |  |  | 1.004 (1.001, 1.008) ** | 0.007 |  |
| Yes | 0.078 (0.032, 0.124) ** | 0.001 |  |  | 1.006 (0.996, 1.016) | 0.236 |  |
| **Cardiovascular disease** |  |  | 0.893 |  |  |  | 0.332 |
| No | 0.044 (0.030, 0.059) *** | <0.001 |  |  | 1.005 (1.002, 1.008) ** | 0.002 |  |
| Yes | 0.041 (-0.017, 0.098) | 0.164 |  |  | 0.999 (0.987, 1.011) | 0.855 |  |
| **COPD, %** |  |  | 0.486 |  |  |  | 0.013 |
| No | 0.046 (0.031, 0.061) *** | <0.001 |  |  | 1.006 (1.003, 1.01) *** | <0.001 |  |
| Yes | 0.033 (0.001, 0.066) * | 0.044 |  |  | 0.997 (0.99, 1.004) | 0.343 |  |
| PM_2.5_: Particulate matter 2.5; BMI: body mass index; CNY: Chinese yuan; OR: odds ratio; CI: confidence interval; COPD: chronic obstructive pulmonary disease.  *: *P* _FDR_<0.05; **: *P* _FDR_ <0.01; ***: *P* _FDR_ <0.001. | | | | | | | |

| **Supplementary table 10.** Associations of long-term exposure to 2-year average PM_10_ with phenotypic age (years) and accelerated age**,** stratified by demographic variables in GBCS (N=9527) | | | | | | | |
| --- | --- | --- | --- | --- | --- | --- | --- |
|  | Phenotypic age (years) | | |  | Accelerated age | | |
|  | β (95 % CI) | *P* _FDR_ | P-value for interaction |  | OR (95 % CI) | *P* _FDR_ | P-value for interaction |
| **Age, years** |  |  | 0.502 |  |  |  | 0.545 |
| <65 | 0.018 (0.006, 0.029) ** | 0.003 |  |  | 1.003 (0.999, 1.006) | 0.125 |  |
| ≥65 | 0.023 (0.011, 0.035) *** | <0.001 |  |  | 1.004 (1.001, 1.007) * | 0.018 |  |
| **Sex** |  |  | 0.655 |  |  |  | 0.448 |
| Men | 0.048 (0.030, 0.066) *** | <0.001 |  |  | 1.007 (1.002, 1.011) ** | 0.002 |  |
| Women | 0.043 (0.031, 0.055) *** | <0.001 |  |  | 1.005 (1.002, 1.008) ** | 0.001 |  |
| **Education level** |  |  | 0.980 |  |  |  | 0.599 |
| Primary or below | 0.034 (0.019, 0.049) *** | <0.001 |  |  | 1.005 (1.001, 1.008) ** | 0.006 |  |
| Secondary | 0.032 (0.016, 0.048) *** | <0.001 |  |  | 1.002 (0.999, 1.006) | 0.218 |  |
| College or above | 0.034 (0.001, 0.068) | 0.050 |  |  | 1.002 (0.994, 1.010) | 0.620 |  |
| **Occupation** |  |  | 0.587 |  |  |  | 0.855 |
| Manual | 0.034 (0.015, 0.053) *** | <0.001 |  |  | 1.004 (1.000, 1.008) | 0.050 |  |
| Non-manual | 0.037 (0.024, 0.05) *** | <0.001 |  |  | 1.003 (1.000, 1.006) * | 0.040 |  |
| Other | 0.009 (-0.043, 0.061) | 0.730 |  |  | 1.001 (0.990, 1.013) | 0.851 |  |
| **Family income, CNY/year** |  |  | 0.853 |  |  |  | 0.867 |
| <10,000 | 0.052 (0.014, 0.091) ** | 0.008 |  |  | 1.001 (0.992, 1.010) | 0.858 |  |
| 10,000–29,999 | 0.033 (0.015, 0.051) *** | <0.001 |  |  | 1.002 (0.998, 1.006) | 0.266 |  |
| 30,000–49,999 | 0.025 (-0.001, 0.052) | 0.058 |  |  | 1.003 (0.997, 1.009) | 0.334 |  |
| ≥50,000 | 0.038 (0.006, 0.071) * | 0.022 |  |  | 1.006 (0.999, 1.013) | 0.112 |  |
| Don’t know | 0.032 (0.014, 0.050) *** | <0.001 |  |  | 1.004 (1.000, 1.008) * | 0.039 |  |
| **Smoking status** |  |  | 0.002 |  |  |  | 0.482 |
| Never | 0.038 (0.026, 0.049) *** | <0.001 |  |  | 1.004 (1.002, 1.007) ** | 0.001 |  |
| Former | 0.084 (0.054, 0.114) *** | <0.001 |  |  | 1.007 (1.000, 1.014) * | 0.049 |  |
| Current | 0.004 (-0.027, 0.035) | 0.813 |  |  | 1.001 (0.993, 1.008) | 0.849 |  |
| **Alcohol use** |  |  | 0.623 |  |  |  | 0.845 |
| Never | 0.037 (0.025, 0.048) *** | <0.001 |  |  | 1.003 (1.000, 1.005) * | 0.024 |  |
| Former | 0.019 (-0.053, 0.090) | 0.606 |  |  | 1.005 (0.990, 1.022) | 0.502 |  |
| Current | 0.023 (-0.004, 0.051) | 0.093 |  |  | 1.005 (0.999, 1.011) | 0.131 |  |
| **BMI, kg/m^2^** |  |  | 0.384 |  |  |  | 0.346 |
| <18.5 | 0.009 (-0.041, 0.058) | 0.730 |  |  | 0.993 (0.981, 1.004) | 0.217 |  |
| 18.5–24.9 | 0.035 (0.022, 0.049) *** | <0.001 |  |  | 1.003 (1.000, 1.006) | 0.065 |  |
| 25.0–27.4 | 0.022 (-0.001, 0.045) | 0.061 |  |  | 1.004 (0.999, 1.009) | 0.092 |  |
| ≥27.5 | 0.049 (0.019, 0.078) ** | 0.001 |  |  | 1.003 (0.997, 1.01) | 0.320 |  |
| **Physical activity** |  |  | 0.076 |  |  |  | 0.606 |
| Inactive | 0.111 (0.015, 0.206) * | 0.025 |  |  | 1.009 (0.991, 1.027) | 0.343 |  |
| Moderate | 0.038 (0.020, 0.056) *** | <0.001 |  |  | 1.004 (1.000, 1.008) * | 0.039 |  |
| Active | 0.025 (0.012, 0.038) *** | <0.001 |  |  | 1.002 (0.999, 1.005) | 0.167 |  |
| **Arthritis** |  |  | 0.452 |  |  |  | 0.332 |
| No | 0.035 (0.023, 0.047) *** | <0.001 |  |  | 1.002 (1.000, 1.005) | 0.079 |  |
| Yes | 0.026 (0.004, 0.048) * | 0.023 |  |  | 1.005 (1.000, 1.010) * | 0.040 |  |
| **Household air pollution** |  |  | 0.312 |  |  |  | 0.485 |
| No | 0.034 (0.023, 0.044) *** | <0.001 |  |  | 1.003 (1.001, 1.005) ** | 0.009 |  |
| Yes | 0.084 (-0.02, 0.187) | 0.115 |  |  | 1.011 (0.990, 1.032) | 0.322 |  |
| **Passive smoking exposure** |  |  | 0.528 |  |  |  | 0.589 |
| <2 years of 40 h per week | 0.046 (0.021, 0.072) *** | <0.001 |  |  | 1.005 (0.999, 1.011) | 0.074 |  |
| 2–5 years of 40 h per week | 0.039 (0.010, 0.069) ** | 0.009 |  |  | 1.003 (0.997, 1.010) | 0.346 |  |
| >5 years of 40 h per week | 0.045 (0.022, 0.068) *** | <0.001 |  |  | 1.006 (1.001, 1.011) * | 0.022 |  |
| Not reported | 0.028 (0.014, 0.043) *** | <0.001 |  |  | 1.002 (0.999, 1.005) | 0.177 |  |
| **Diabetes** |  |  | 0.592 |  |  |  | 0.910 |
| No | 0.032 (0.021, 0.042) *** | <0.001 |  |  | 1.003 (1.001, 1.005) * | 0.016 |  |
| Yes | 0.041 (0.003, 0.080) * | 0.036 |  |  | 1.004 (0.995, 1.012) | 0.424 |  |
| **Hypertension** |  |  | 0.268 |  |  |  | 0.185 |
| No | 0.030 (0.017, 0.042) *** | <0.001 |  |  | 1.002 (0.999, 1.005) | 0.151 |  |
| Yes | 0.042 (0.023, 0.062) *** | <0.001 |  |  | 1.005 (1.001, 1.010) ** | 0.009 |  |
| **Dyslipidaemia** |  |  | 0.109 |  |  |  | 0.571 |
| No | 0.031 (0.020, 0.042) *** | <0.001 |  |  | 1.003 (1.001, 1.005) * | 0.015 |  |
| Yes | 0.061 (0.026, 0.097) ** | 0.001 |  |  | 1.005 (0.998, 1.013) | 0.172 |  |
| **Cardiovascular disease** |  |  | 0.945 |  |  |  | 0.267 |
| No | 0.034 (0.024, 0.045) *** | <0.001 |  |  | 1.004 (1.001, 1.006) ** | 0.004 |  |
| Yes | 0.033 (-0.011, 0.077) | 0.142 |  |  | 0.998 (0.989, 1.007) | 0.704 |  |
| **COPD, %** |  |  | 0.760 |  |  |  | 0.029 |
| No | 0.035 (0.023, 0.046) *** | <0.001 |  |  | 1.004 (1.002, 1.007) ** | 0.001 |  |
| Yes | 0.031 (0.005, 0.056) * | 0.017 |  |  | 0.998 (0.992, 1.003) | 0.42 |  |
| PM_10_: Particulate matter 10; BMI: body mass index; CNY: Chinese yuan; OR: odds ratio; CI: confidence interval; COPD: chronic obstructive pulmonary disease.  *: *P* _FDR_<0.05; **: *P* _FDR_ <0.01; ***: *P* _FDR_ <0.001. | | | | | | | |

| **Supplementary table 11.** Associations of long-term exposure to 1-year average PM_2.5_ and PM_10_ with accelerated age, stratified by PDI and PHD in GBCS (N=9527) | | | |
| --- | --- | --- | --- |
| 1-year average |  | Accelerated age, OR (95 % CI) | |
|  |  | Crude model | Adjusted model ^a^ |
| PM_2.5_ |  |  |  |
| Per 1 μg/m^3^ increase in PM_2.5_ |  | 1.008 (1.004, 1.012) *** | 1.010 (1.006, 1.015) *** |
| Stratified by PDI |  |  |  |
| Lower PDI |  |  |  |
| Per 1 μg/m^3^ increase in PM_2.5_ |  | 1.013 (1.007, 1.019) *** | 1.013 (1.006, 1.019) *** |
| Higher PDI |  |  |  |
| Per 1 μg/m^3^ increase in PM_2.5_ |  | 1.003 (1.000, 1.008) * | 1.009 (1.003, 1.015) ** |
| Stratified by PHD |  |  |  |
| Lower PHD |  |  |  |
| Per 1 μg/m^3^ increase in PM_2.5_ |  | 1.009 (1.003, 1.015) ** | 1.011 (1.004, 1.017) *** |
| Higher PHD |  |  |  |
| Per 1 μg/m^3^ increase in PM_2.5_ |  | 1.007 (1.001, 1.012) * | 1.009 (1.004, 1.016) ** |
| PM_10_ |  |  |  |
| Per 1 μg/m^3^ increase in PM_10_ |  | 1.006 (1.003, 1.009) *** | 1.008 (1.004, 1.011) *** |
| Stratified by PDI |  |  |  |
| Lower PDI |  |  |  |
| Per 1 μg/m^3^ increase in PM_10_ |  | 1.010 (1.005, 1.014) *** | 1.010 (1.005, 1.015) *** |
| Higher PDI |  |  |  |
| Per 1 μg/m^3^ increase in PM_10_ |  | 1.002 (1.000, 1.006) * | 1.006 (1.002, 1.011) ** |
| Stratified by PHD |  |  |  |
| Lower PHD |  |  |  |
| Per 1 μg/m^3^ increase in PM_10_ |  | 1.007 (1.002, 1.011) * | 1.009 (1.004, 1.013) ** |
| Higher PHD |  |  |  |
| Per 1 μg/m^3^ increase in PM_10_ |  | 1.005 (1.001, 1.009) ** | 1.007 (1.003, 1.012) *** |

PM_2.5_: Particulate matter 2.5; PM_10_: Particulate matter 10; PDI: plant-based diets index; PHD: planetary-health diets; OR: odds ratio; CI: confidence interval.

Accelerated age: accelerated age was dichotomized into two categories: presence or absence of accelerated aging, with values greater than 5 indicating accelerated aging.

^a^: Adjusted for age, sex, education level, occupation, family income, smoking status, alcohol use, BMI, physical activity, household air pollution, passive smoking exposure, temperature, humidity, O_3_, arthritis, diabetes, hypertension, dyslipidaemia, cardiovascular disease, COPD.

*: *P* _FDR_<0.05; **: *P* _FDR_ <0.01; ***: *P* _FDR_ <0.001.

**Supplementary table 12.** Characteristics of participants in the South China Cohort

| Characteristics | Total  (N=2023) | **1-year average PM_2.5_** | | | |  | **1-year average PM_10_** | | | |
| --- | --- | --- | --- | --- | --- | --- | --- | --- | --- | --- |
|  |  | Tertile 1  (N=675) | Tertile 2  (N=674) | Tertile 3  (N=674) | P value |  | Tertile 1  (N=675) | Tertile 2  (N=674) | Tertile 3  (N=674) | P value |
| **O_3_, μg/m³, Median (Q1, Q3)** | 93.9 (92.8, 95.1) | 94.1 (92.9, 95.1) | 94.5 (93.4, 95.4) | 93.1 (92.6, 94.4) | < 0.001 |  | 94.2 (92.9, 95.1) | 94.2 (93.1, 95.3) | 93.1 (92.6, 94.5) | < 0.001 |
| **NO_2_, μg/m³, Median (Q1, Q3)** | 49.5 (47.1, 49.9) | 49.5 (47, 50) | 49.3 (43.9, 49.5) | 49.6 (49.3, 50.3) | < 0.001 |  | 49.5 (47.2, 50) | 49.3 (44, 49.5) | 49.7 (49.3, 50.3) | < 0.001 |
| **Temperature, °C, Median (Q1, Q3)** | 23.1 (23, 23.1) | 23.1 (23, 23.1) | 23.1 (23, 23.1) | 23.1 (23.1, 23.1) | < 0.001 |  | 23.1 (23, 23.1) | 23.1 (23, 23.1) | 23.1 (23.1, 23.1) | < 0.001 |
| **Humidity, %, Median (Q1, Q3)** | 73.6 (73.2, 73.6) | 73.6 (73.2, 73.6) | 73.6 (73.5, 73.6) | 73.6 (73, 73.6) | < 0.001 |  | 73.6 (73.2, 73.6) | 73.6 (73.5, 73.6) | 73.5 (73, 73.6) | < 0.001 |
| **Age, years** |  |  |  |  | 0.034 |  |  |  |  | 0.586 |
| <65 | 1736 (85.8) | 574 (85) | 565 (83.8) | 597 (88.6) |  |  | 576 (85.3) | 574 (85.2) | 586 (86.9) |  |
| ≥65 | 287 (14.2) | 101 (15) | 109 (16.2) | 77 (11.4) |  |  | 99 (14.7) | 100 (14.8) | 88 (13.1) |  |
| **Sex** |  |  |  |  | 0.989 |  |  |  |  | 0.996 |
| Men | 623 (30.8) | 207 (30.7) | 209 (31) | 207 (30.7) |  |  | 207 (30.7) | 208 (30.9) | 208 (30.9) |  |
| Women | 1400 (69.2) | 468 (69.3) | 465 (69) | 467 (69.3) |  |  | 468 (69.3) | 466 (69.1) | 466 (69.1) |  |
| **Education level** |  |  |  |  | 0.006 |  |  |  |  | 0.044 |
| Primary or below | 119 (5.9) | 42 (6.2) | 45 (6.7) | 32 (4.7) |  |  | 41 (6.1) | 38 (5.6) | 40 (5.9) |  |
| Secondary | 1510 (74.6) | 525 (77.8) | 472 (70) | 513 (76.1) |  |  | 520 (77) | 479 (71.1) | 511 (75.8) |  |
| College or above | 394 (19.5) | 108 (16) | 157 (23.3) | 129 (19.1) |  |  | 114 (16.9) | 157 (23.3) | 123 (18.2) |  |
| **Marital status** |  |  |  |  | 0.1 |  |  |  |  | 0.128 |
| Never married | 17 (0.8) | 5 (0.8) | 5 (0.7) | 7 (1) |  |  | 5 (0.7) | 5 (0.7) | 7 (1) |  |
| Married | 1882 (93) | 634 (93.9) | 614 (91.1) | 634 (94.1) |  |  | 638 (94.5) | 615 (91.2) | 629 (93.3) |  |
| Separated/divorced/widowed | 124 (6.2) | 36 (5.3) | 55 (8.2) | 33 (4.9) |  |  | 32 (4.7) | 54 (8) | 38 (5.6) |  |
| **Occupation** |  |  |  |  | 0.009 |  |  |  |  | < 0.001 |
| Manual | 567 (28) | 195 (28.9) | 188 (27.9) | 184 (27.3) |  |  | 197 (29.2) | 182 (27) | 188 (27.9) |  |
| Non-manual | 550 (27.2) | 208 (30.8) | 185 (27.4) | 157 (23.3) |  |  | 219 (32.4) | 175 (26) | 156 (23.1) |  |
| Other | 906 (44.8) | 272 (40.3) | 301 (44.7) | 333 (49.4) |  |  | 259 (38.4) | 317 (47) | 330 (49) |  |
| **Family income, CNY/year** |  |  |  |  | 0.158 |  |  |  |  | 0.17 |
| <50,000 | 444 (21.9) | 143 (21.2) | 152 (22.6) | 149 (22.1) |  |  | 145 (21.5) | 143 (21.2) | 156 (23.1) |  |
| 50,000–79,999 | 662 (32.7) | 197 (29.2) | 226 (33.5) | 239 (35.5) |  |  | 197 (29.2) | 238 (35.3) | 227 (33.7) |  |
| ≥80,000 | 886 (43.8) | 322 (47.7) | 287 (42.6) | 277 (41.1) |  |  | 320 (47.4) | 283 (42) | 283 (42) |  |
| Don’t know | 31 (1.5) | 13 (1.9) | 9 (1.3) | 9 (1.3) |  |  | 13 (1.9) | 10 (1.5) | 8 (1.2) |  |
| **Smoking status** |  |  |  |  | 0.342 |  |  |  |  | 0.95 |
| Never | 1658 (82) | 543 (80.4) | 561 (83.2) | 554 (82.2) |  |  | 549 (81.3) | 556 (82.5) | 553 (82) |  |
| Former | 205 (10.1) | 68 (10.1) | 70 (10.4) | 67 (9.9) |  |  | 70 (10.4) | 69 (10.2) | 66 (9.8) |  |
| Current | 160 (7.9) | 64 (9.5) | 43 (6.4) | 53 (7.9) |  |  | 56 (8.3) | 49 (7.3) | 55 (8.2) |  |
| **Alcohol use** |  |  |  |  | 0.001 |  |  |  |  | 0.064 |
| Never | 1624 (80.3) | 557 (82.5) | 529 (78.5) | 538 (79.8) |  |  | 547 (81) | 536 (79.5) | 541 (80.3) |  |
| Former | 249 (12.3) | 90 (13.3) | 87 (12.9) | 72 (10.7) |  |  | 93 (13.8) | 81 (12) | 75 (11.1) |  |
| Current | 150 (7.4) | 28 (4.1) | 58 (8.6) | 64 (9.5) |  |  | 35 (5.2) | 57 (8.5) | 58 (8.6) |  |
| **BMI, kg/m^2^** |  |  |  |  | 0.911 |  |  |  |  | 0.752 |
| <18.5 | 82 (4.1) | 28 (4.1) | 26 (3.9) | 28 (4.2) |  |  | 27 (4) | 26 (3.9) | 29 (4.3) |  |
| 18.5–24.9 | 1393 (68.9) | 468 (69.3) | 472 (70) | 453 (67.2) |  |  | 479 (71) | 451 (66.9) | 463 (68.7) |  |
| 25.0–27.4 | 365 (18) | 121 (17.9) | 113 (16.8) | 131 (19.4) |  |  | 115 (17) | 131 (19.4) | 119 (17.7) |  |
| ≥27.5 | 183 (9) | 58 (8.6) | 63 (9.3) | 62 (9.2) |  |  | 54 (8) | 66 (9.8) | 63 (9.3) |  |
| **Arthritis** |  |  |  |  | 0.734 |  |  |  |  | 0.264 |
| No | 1885 (93.2) | 633 (93.8) | 625 (92.7) | 627 (93) |  |  | 630 (93.3) | 635 (94.2) | 620 (92) |  |
| Yes | 138 (6.8) | 42 (6.2) | 49 (7.3) | 47 (7) |  |  | 45 (6.7) | 39 (5.8) | 54 (8) |  |
| **Diabetes** |  |  |  |  | 0.002 |  |  |  |  | 0.008 |
| No | 1669 (82.5) | 529 (78.4) | 568 (84.3) | 572 (84.9) |  |  | 534 (79.1) | 559 (82.9) | 576 (85.5) |  |
| Yes | 354 (17.5) | 146 (21.6) | 106 (15.7) | 102 (15.1) |  |  | 141 (20.9) | 115 (17.1) | 98 (14.5) |  |
| **Hypertension** |  |  |  |  | 0.331 |  |  |  |  | 0.383 |
| No | 1447 (71.5) | 469 (69.5) | 486 (72.1) | 492 (73) |  |  | 479 (71) | 473 (70.2) | 495 (73.4) |  |
| Yes | 576 (28.5) | 206 (30.5) | 188 (27.9) | 182 (27) |  |  | 196 (29) | 201 (29.8) | 179 (26.6) |  |
| **Dyslipidaemia** |  |  |  |  | 0.072 |  |  |  |  | 0.179 |
| No | 1394 (68.9) | 443 (65.6) | 479 (71.1) | 472 (70) |  |  | 447 (66.2) | 475 (70.5) | 472 (70) |  |
| Yes | 629 (31.1) | 232 (34.4) | 195 (28.9) | 202 (30) |  |  | 228 (33.8) | 199 (29.5) | 202 (30) |  |
| **Cardiovascular disease** |  |  |  |  | 0.004 |  |  |  |  | 0.089 |
| No | 1651 (80.3) | 542 (76.7) | 544 (80.7) | 565 (83.8) |  |  | 530 (78.5) | 544 (80.7) | 561 (83.2) |  |
| Yes | 404 (19.7) | 165 (23.3) | 130 (19.3) | 109 (16.2) |  |  | 145 (21.5) | 130 (19.3) | 113 (16.8) |  |

Q1: Quartile 1; Q3: Quartile 3; SD: Standard deviation; PM_2.5_: Particulate matter 2.5; PM_10_: Particulate matter 10; BMI: body mass index; CNY: Chinese yuan; O_3_: ozone; NO_2_: nitrogen dioxide.


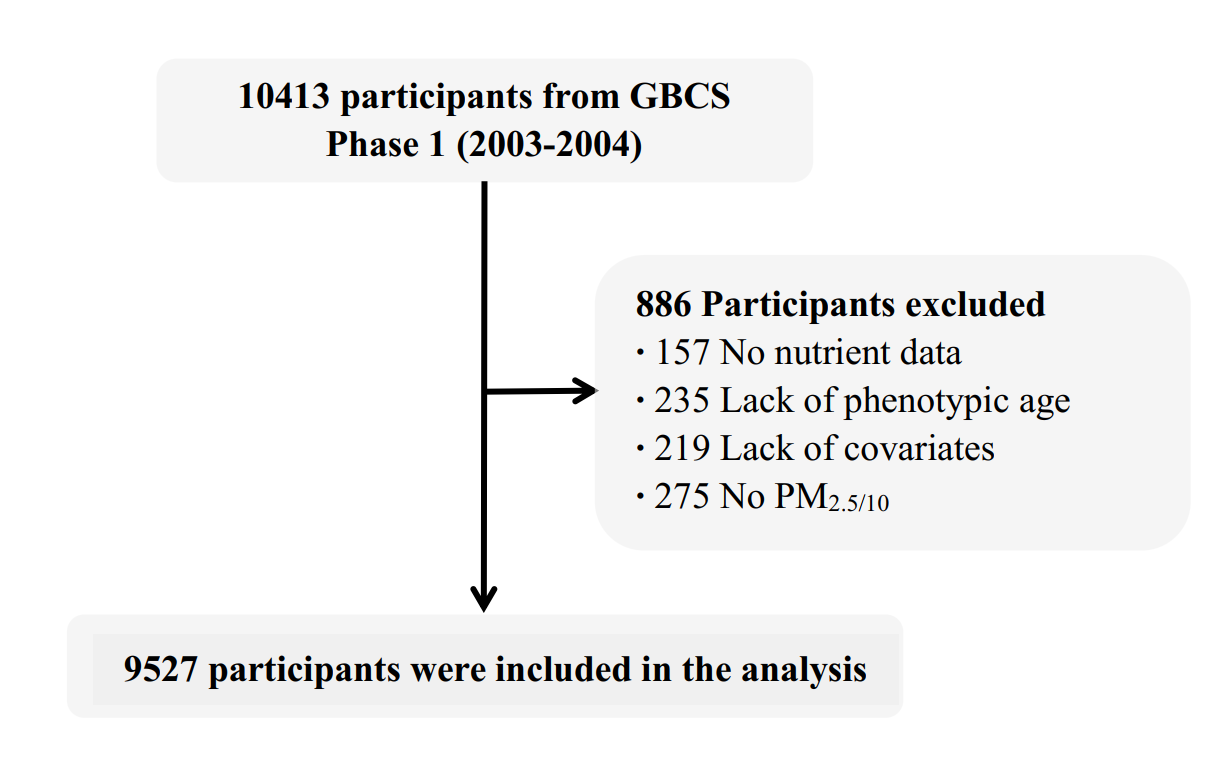


**Supplementary Figure 1.** Study sample selection process in the GBCS


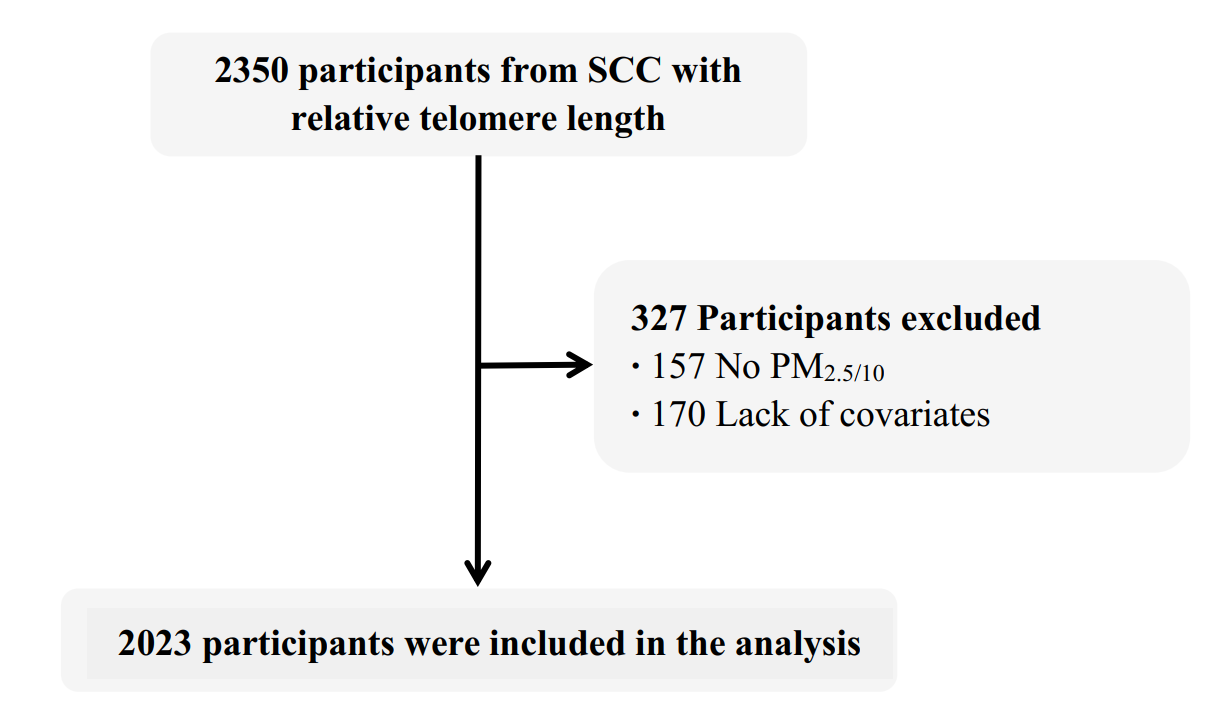


**Supplementary Figure 2.** Study sample selection process in the SCC


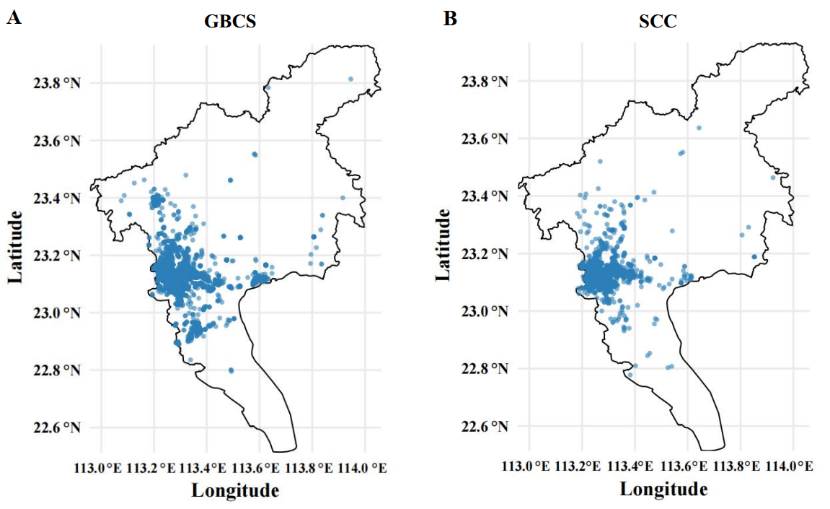


**Supplementary Figure 3.** Spatial distribution of baseline residential address of study participants in Guangzhou, China. The maps illustrate the geographical distribution of residences for participants included in the current study. (A) GBCS, n =9,527; (B) SCC, n = 2,023. Blue dot represents geocoded baseline residences; black outline shows the administrative boundary of Guangzhou. Latitude and longitude are provided on the Y and X axes, respectively


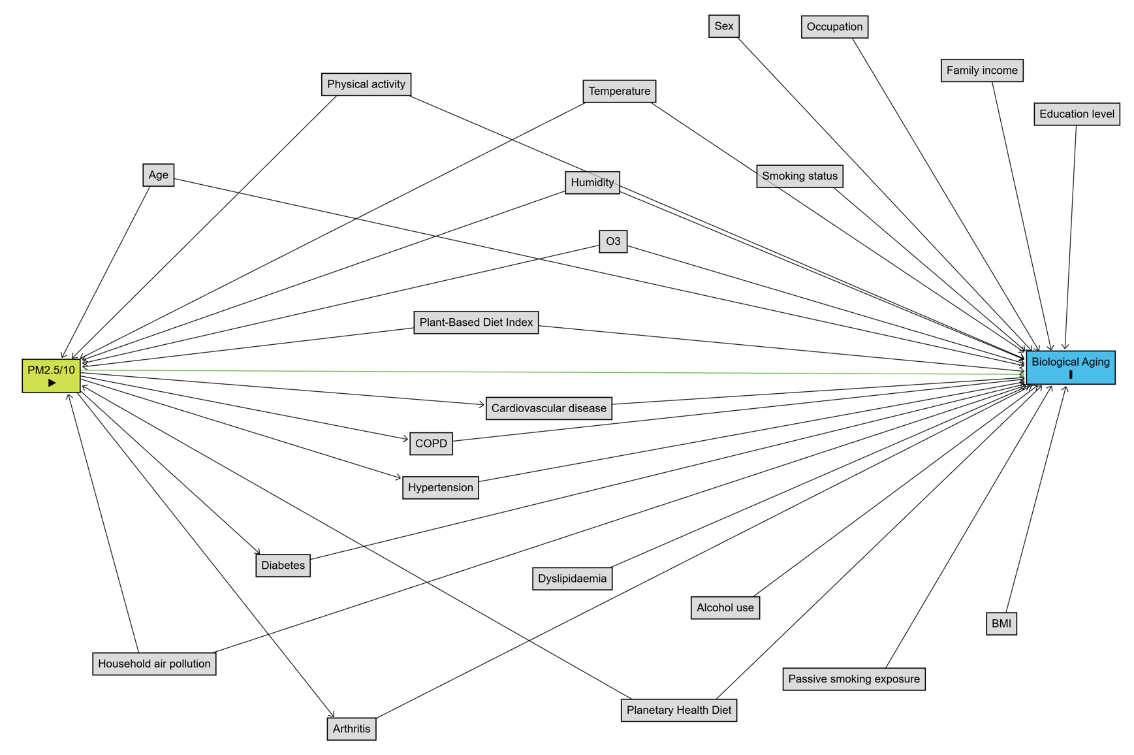


**Supplementary Figure 4.** Directed Acyclic Graph


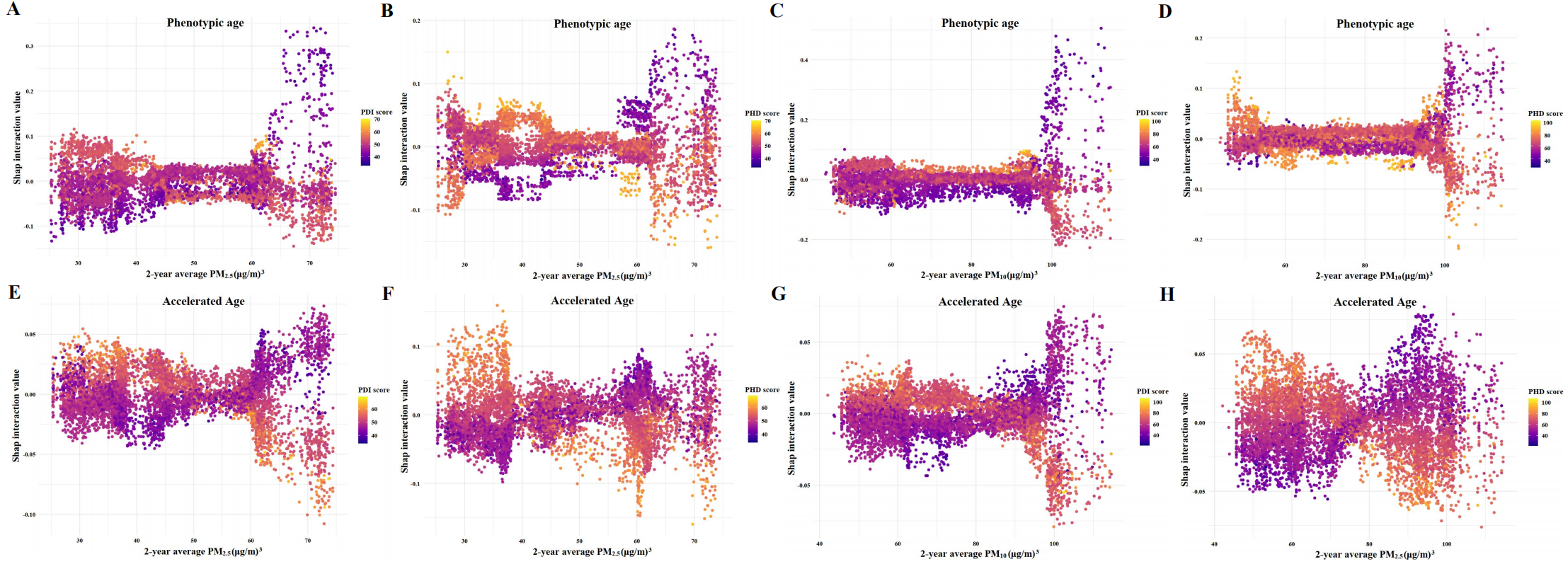


**Supplementary Figure 5.** SHAP interaction plots for 2-year average PM_2.5_ and PM_10_ exposure (μg/m³) and individual PDI/PHD scores in relation to phenotypic age (years) and accelerated age in the GBCS

Note: The color gradient represents the value of the interaction feature (PDI or PHD score), ranging from low (purple) to high (yellow). Panels display interactions for Phenotypic Age (A–D) and Accelerated Age (E–H): (A, E) PM_2.5_ and PDI scores; (B, F) PM_2.5_ and PHD scores; (C, G) PM_10_ and PDI scores; (D, H) PM_10_ and PHD scores. Models were adjusted for age, sex, education level, occupation, family income, smoking status, alcohol use, BMI, physical activity, household air pollution, passive smoking exposure, temperature, humidity, O_3_, arthritis, diabetes, hypertension, dyslipidaemia, cardiovascular disease, COPD.

Abbreviations: SHAP, SHapley Additive exPlanations; PM, particulate matter; PDI, Plant-Based Diet Index; PHD, Planetary Health Diet; GBCS, Guangzhou Biobank Cohort Study; BMI, body mass index; COPD, chronic obstructive pulmonary disease; O_3_, ozone.


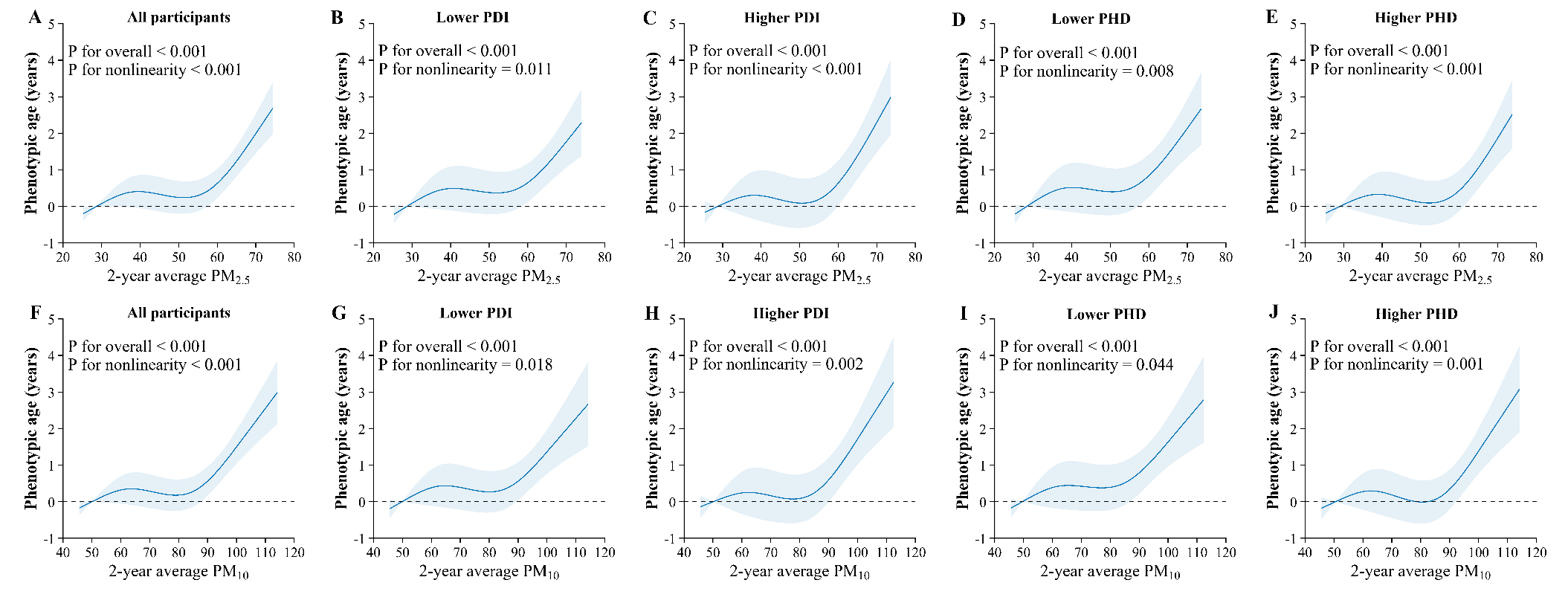


**Supplementary Figure 6.** Associations of 2-year average PM_2.5_ and PM_10_ concentrations (μg/m³) with phenotypic age (years), stratified by PDI and PHD in the GBCS

Note: Panels A–E show associations with PM_2.5_, and panels F–J show associations with PM_10_. The first panels (A, F) represent the total study population, while the subsequent panels show results stratified by lower and higher levels of PDI and PHD. Solid lines represent β coefficients, and shaded areas indicate 95% confidence intervals (CIs). Models were adjusted for age, sex, education level, occupation, family income, smoking status, alcohol use, BMI, physical activity, household air pollution, passive smoking exposure, temperature, humidity, O_3_, arthritis, diabetes, hypertension, dyslipidaemia, cardiovascular disease, COPD.

Abbreviations: PM, particulate matter; PDI, Plant-Based Diet Index; PHD, Planetary Health Diet; GBCS, Guangzhou Biobank Cohort Study; BMI, body mass index; COPD, chronic obstructive pulmonary disease; CI, confidence interval; O_3_, ozone.


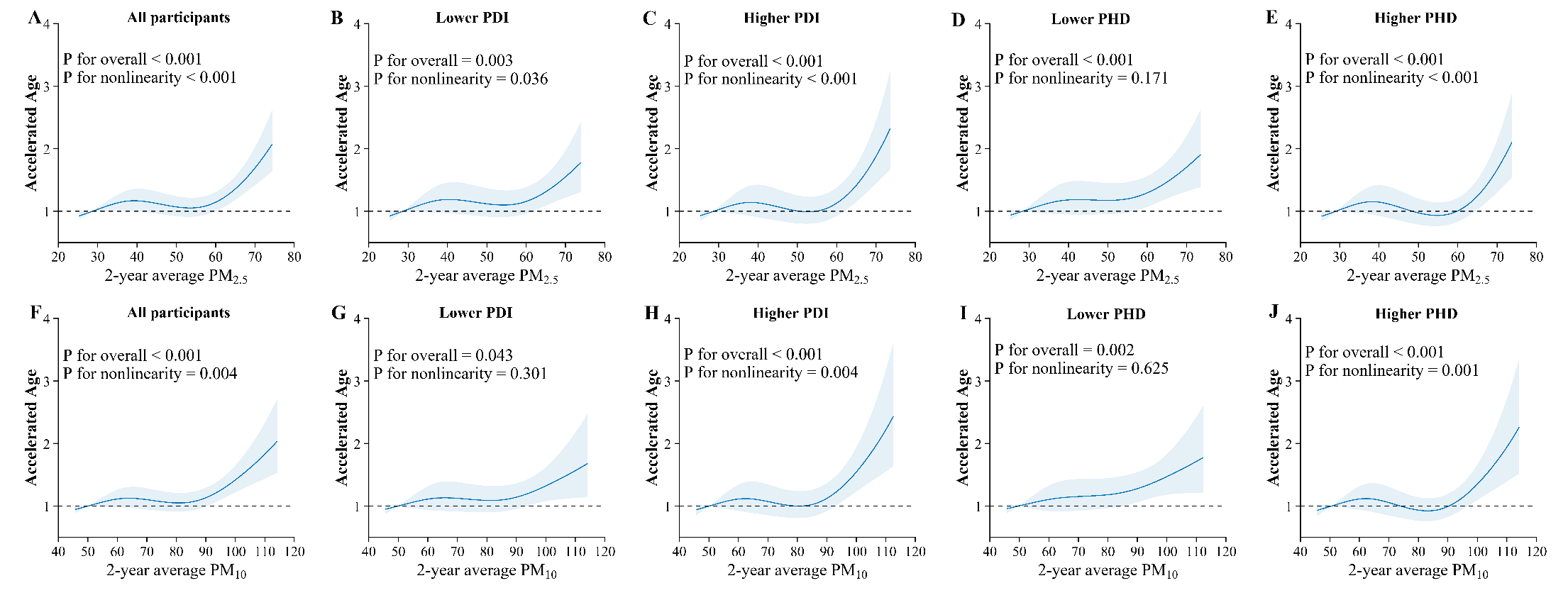


**Supplementary Figure 7.** Associations of 2-year average PM_2.5_ and PM_10_ concentrations (μg/m³) with accelerated age, stratified by PDI and PHD in the GBCS

Note: Panels A–E show associations with PM_2.5_, and panels F–J show associations with PM_10_. Panels A and F represent the total study population, while the remaining panels display results stratified by lower and higher levels of PDI and PHD. Solid lines represent odds ratios (ORs), and shaded areas indicate 95% confidence intervals (CIs). Models were adjusted for age, sex, education level, occupation, family income, smoking status, alcohol use, BMI, physical activity, household air pollution, passive smoking exposure, temperature, humidity, O_3_, arthritis, diabetes, hypertension, dyslipidaemia, cardiovascular disease, COPD.

Abbreviations: PM, particulate matter; OR, odds ratio; CI, confidence interval; PDI, Plant-Based Diet Index; PHD, Planetary Health Diet; GBCS, Guangzhou Biobank Cohort Study; BMI, body mass index; COPD, chronic obstructive pulmonary disease; O_3_, ozone.


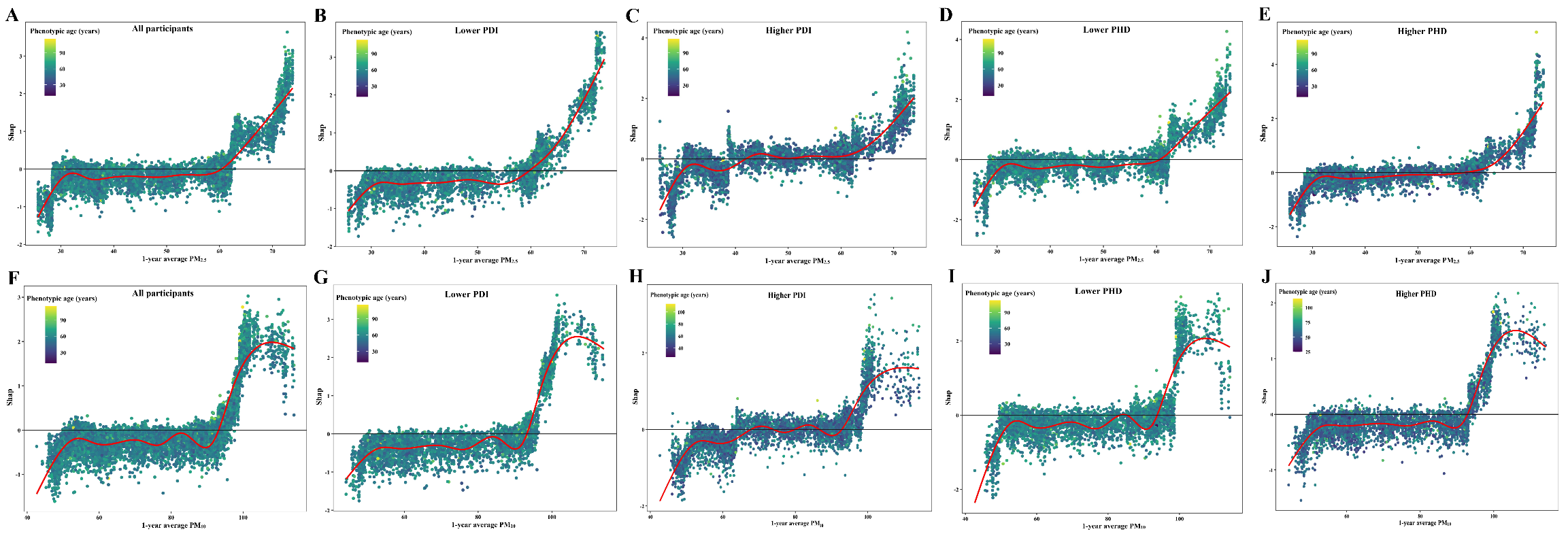


**Supplementary Figure 8.** Associations of 1-year average PM_2.5_ and PM_10_ exposure with the SHAP values for phenotypic age (years), stratified by PDI and PHD

Note: Panels A–E depict the association between 1-year average PM_2.5_ exposure and the SHAP values for phenotypic age, while panels F–J show the association for 1-year average PM_10_ exposure. Panels A and F represent data for all participants, while the remaining panels (B–E and G–J) show results stratified by lower and higher levels of PDI and PHD. The color scale represents phenotypic age in years. The red lines depict the fitted association between PM exposure and SHAP values, indicating the contribution of PM to phenotypic age.

Abbreviations: PM, particulate matter; SHAP, SHapley Additive exPlanations; PDI, Plant-Based Diet Index; PHD, Planetary Health Diet.


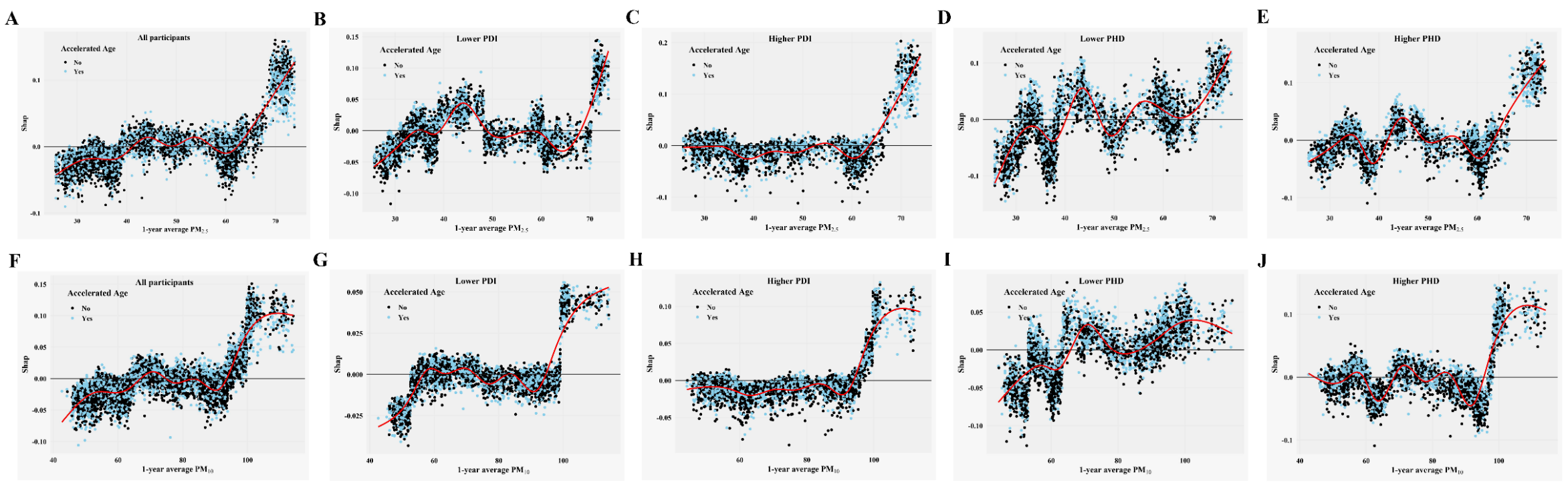


**Supplementary Figure 9.** Associations of 1-year average PM_2.5_ and PM_10_ exposure with the SHAP values for accelerated age, stratified by PDI and PHD

Note: Panels A–E show the association between 1-year average PM_2.5_ and the SHAP values for accelerated age, while panels F–J display the association with 1-year average PM_10_. Panels A and F represent data for all participants, while the remaining panels (B–E and G–J) show results stratified by lower and higher levels of PDI and PHD. Black dots represent participants without accelerated age, and blue dots represent participants with accelerated age. The red lines depict the fitted association between PM exposure and SHAP values, indicating the contribution of PM to accelerated age.

Abbreviations: PM, particulate matter; SHAP, SHapley Additive exPlanations; PDI, Plant-Based Diet Index; PHD, Planetary Health Diet.


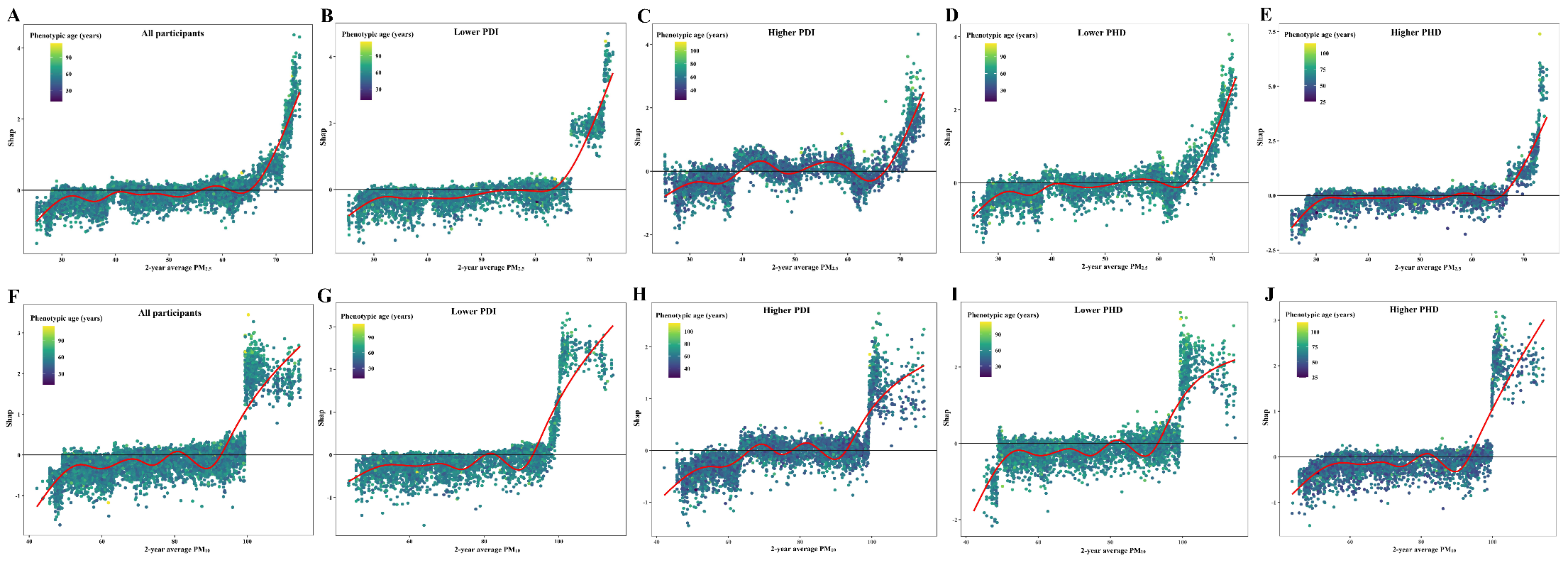


**Supplementary Figure 10.** Associations of 2-year average PM_2.5_ and PM_10_ exposure with the SHAP values for phenotypic age (years), stratified by PDI and PHD

Note: Panels A–E depict the association between 2-year average PM_2.5_ exposure and the SHAP values for phenotypic age, while panels F–J show the association for 2-year average PM_10_ exposure. Panels A and F represent data for all participants, while the remaining panels (B–E and G–J) show results stratified by lower and higher levels of PDI and PHD. The color scale represents phenotypic age in years. The red lines depict the fitted association between PM exposure and SHAP values, indicating the contribution of PM to phenotypic age.

Abbreviations: PM, particulate matter; SHAP, SHapley Additive exPlanations; PDI, Plant-Based Diet Index; PHD, Planetary Health Diet.


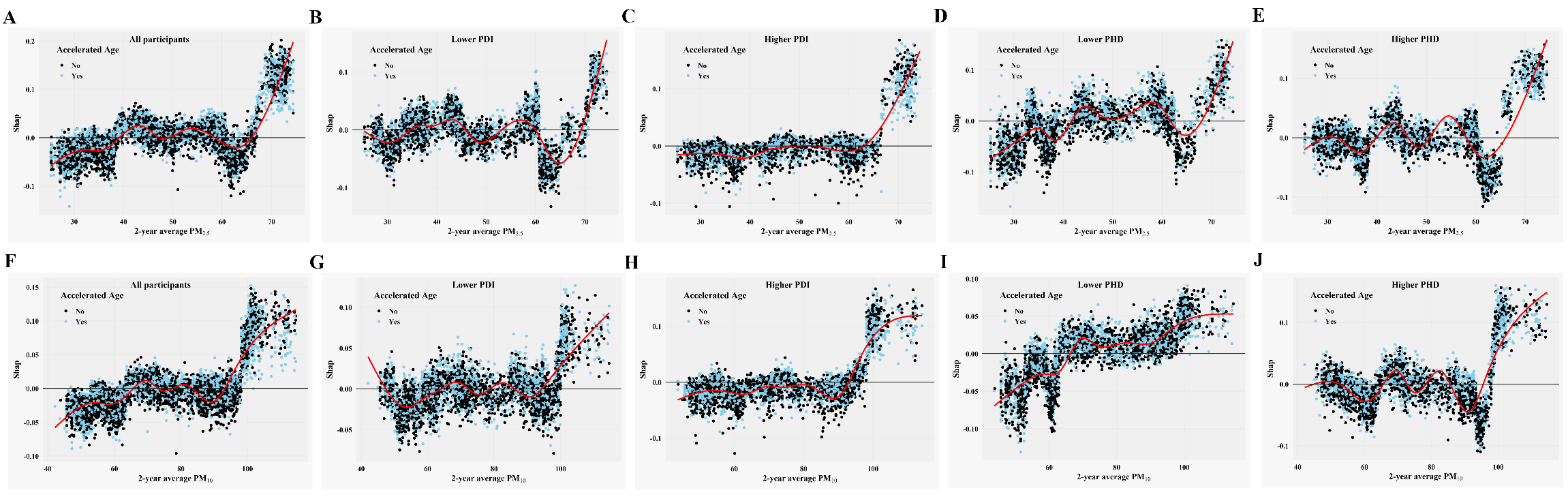


**Supplementary Figure 11.** Associations of 2-year average PM_2.5_ and PM_10_ exposure with the SHAP values for accelerated age, stratified by PDI and PHD

Note: Panels A–E show the association between 2-year average PM_2.5_ and the SHAP values for accelerated age, while panels F–J display the association with 2-year average PM_10_. Panels A and F represent data for all participants, while the remaining panels (B–E and G–J) show results stratified by lower and higher levels of PDI and PHD. Black dots represent participants without accelerated age, and blue dots represent participants with accelerated age. The red lines depict the fitted association between PM exposure and SHAP values, indicating the contribution of PM to accelerated age.

Abbreviations: PM, particulate matter; SHAP, SHapley Additive exPlanations; PDI, Plant-Based Diet Index; PHD, Planetary Health Diet.


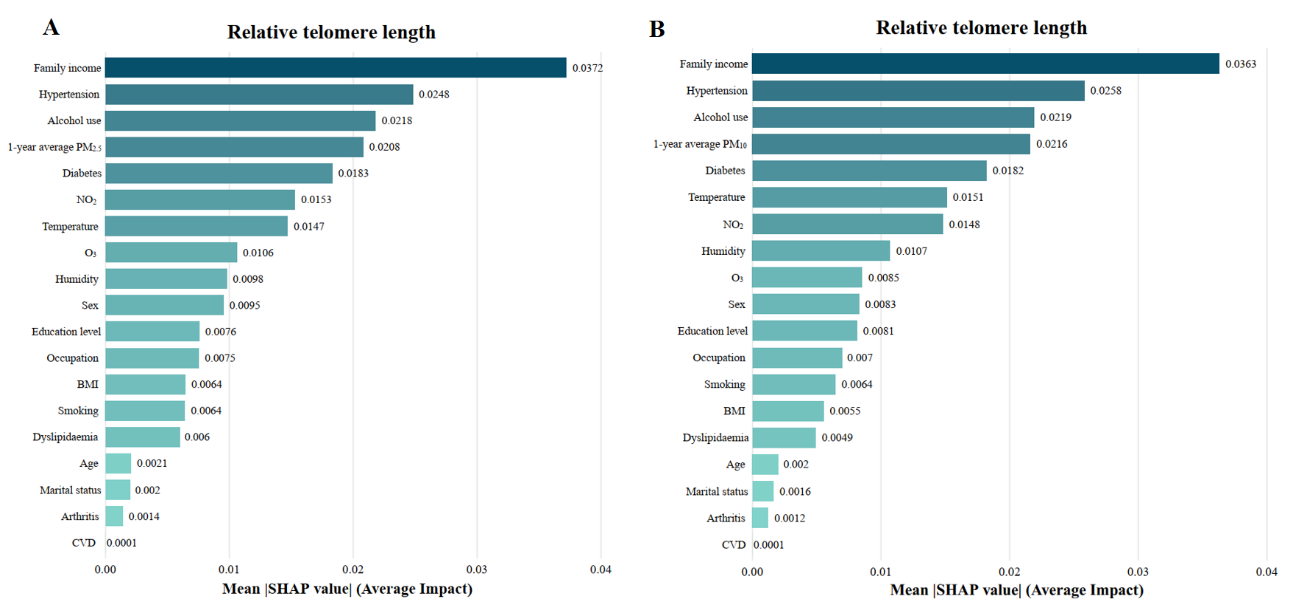


**Supplementary Figure 12.** SHAP summary plots of variable importance for predicting relative telomere length (RTL) using XGBoost models in SCC.

Note: Panel (A) represents the PM_2.5_ 1-year average model; Panel (B) represents the PM_10_ 1-year average model. Variables are ranked in descending order based on their mean absolute SHAP values (|SHAP|), quantifying the global contribution of each feature to the model’s output. The color gradient, from light pink to dark blue, denotes the magnitude of SHAP values, where darker shades signify higher predictive importance.


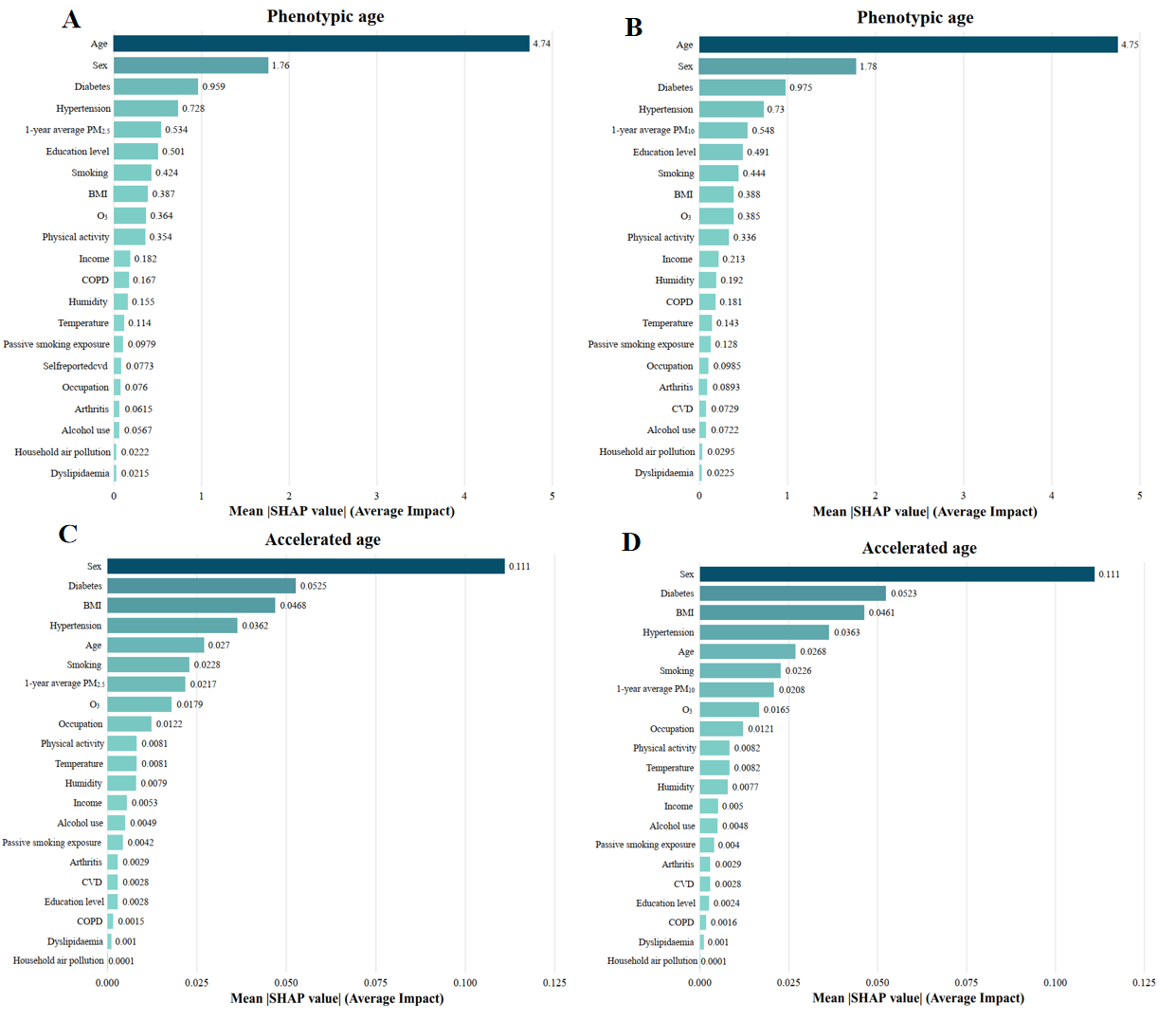


**Supplementary Figure 13.** SHAP summary plots of variable importance for predicting phenotypic age and accelerated age using XGBoost models in GBCS.

Note: Phenotypic age :(A) 1-year average PM_2.5_, (B) 1-year average PM_10_ exposure. Accelerated age :(C) 1-year average PM_2.5_, and (D) 1-year average PM_10_ exposure.Variables are ranked in descending order based on their mean absolute SHAP values (|SHAP|), quantifying the global contribution of each feature to the model’s output. The color gradient, from light pink to dark blue, denotes the magnitude of SHAP values, where darker shades signify higher predictive importance.
